# Flutamide treatment reveals a relationship between steroidogenic activity of Leydig cells and ultrastructure of their mitochondria

**DOI:** 10.1038/s41598-021-93292-8

**Published:** 2021-07-02

**Authors:** Malgorzata Brzoskwinia, Laura Pardyak, Alicja Kaminska, Wacław Tworzydlo, Anna Hejmej, Sylwia Marek, Szczepan M. Bilinski, Barbara Bilinska

**Affiliations:** 1 Department of Endocrinology, Faculty of Biology, Institute of Zoology and Biomedical Research, Jagiellonian University in Krakow, 30-387 Kraków, Poland; 2 Center of Experimental and Innovative Medicine, University of Agriculture in Krakow, 30-248 Kraków, Poland; 3 Department of Developmental Biology and Invertebrate Morphology, Faculty of Biology, Institute of Zoology and Biomedical Research, Jagiellonian University in Krakow, 30-387 Kraków, Poland

**Keywords:** Cell biology, Physiology, Endocrinology

## Abstract

Our present knowledge on interrelation between morphology/ultrastructure of mitochondria of the Leydig cell and its steroidogenic function is far from satisfactory and needs additional studies. Here, we analyzed the effects of blockade of androgen receptor, triggered by exposure to flutamide, on the expression of steroidogenic proteins (1) and ultrastructure of Leydig cells’ constituents (2). We demonstrated that increase in the expression level of steroidogenic (StAR, CYP11A1, 3β-HSD, and CYP19A1) proteins (and respective mRNAs) in rat testicular tissue as well as elevation of intratesticular sex steroid hormone (testosterone and estradiol) levels observed in treated animals correspond well to morphological alterations of the Leydig cell ultrastructure. Most importantly, up-regulation of steroidogenic proteins’ expression apparently correlates with considerable multiplication of Leydig cell mitochondria and subsequent formation of local mitochondrial networks. Interestingly, we showed also that the above-mentioned processes were associated with elevated transcription of *Drp1* and *Mfn2* genes, encoding proteins implicated in mitochondrial dynamics. Collectively, our studies emphasize the importance of mitochondrial homeostasis to the steroidogenic function of Leydig cells.

## Introduction

Leydig cells represent main cellular constituent of the interstitial compartment of the testis and the major site of the testosterone synthesis. The cytoplasm of Leydig cells (for characteristic features of steroid-producing cells see^1^) contains an abundant smooth endoplasmic reticulum (SER), mitochondria (homogeneously distributed throughout the cytoplasm), and lipid droplets where cholesterol esters are converted to free cholesterol. The enzymes involved in the conversion of cholesterol to testosterone are located in either the mitochondria or the SER^2–5^.

In adult males, the maintenance of testosterone secretion is controlled by the hypothalamic–pituitary–gonadal axis (HPG) and modulated by para- and autocrine factors (for further details see^3,6–8^). Several lines of evidence clearly indicated that for testosterone synthesis Leydig cell mitochondria should be fully functional (see below). For instance, in a series of inquisitive experiments on MA-10 Leydig tumor cells treated with mitochondrial disruptors Allen et al.^9^ demonstrated that maintenance of mitochondrial membrane potential, mitochondrial ATP synthesis, and mitochondrial pH are crucial for acute cAMP-stimulated steroid biosynthesis. Moreover, in vitro experiments on Leydig cells isolated from Brown Norway rats and incubated with mitochondrial toxin myxothiazol (a blocker of electron-transport chain) evidenced an important role of mitochondria in basal and luteinizing hormone (LH)-stimulated testosterone production^10^.

Mitochondria contain their own genome (mitochondrial DNA, mtDNA) and protein synthesis machinery. They are therefore regarded as semi-autonomous organelles. Mitochondria supply cells with energy (in the form of ATP), regulate calcium signaling, and contribute to reactive oxygen species (ROS) production^11,12^. The latter process leads to a loss of metabolic functions, decline in mitochondrial membrane potential and, most importantly, to gradual accumulation of mtDNA mutations. In somatic cells, the effects caused by ROS are prevented by the mechanism termed “mitochondrial homeostasis” or “mitochondrial dynamics”. It includes two opposing processes: mitochondrial fusion and mitochondrial fission (division). Disruption of mitochondrial fission leads to the formation of extensive mitochondrial networks, whereas lowering of mitochondrial fusion results in maintaining mitochondria in the form of small individual organelles^13–16^. Consequently, the actual morphology of mitochondria in a given cell is related to a balance between above mentioned processes. The fusion and fission are mediated by multi-domain GTPases belonging to the superfamily of dynamins (see^17^ for a review). Mitochondrial fusion requires such proteins as mitofusins, Mfn1, Mfn2 and Optic atrophy 1 (Opa1), whereas fission involves a highly conserved dynamin-related protein 1 (Drp1)^18,19^. It is well established that Drp1 exhibits an ability to self-assemble into helical structures encompassing the constriction sites of dividing mitochondria that leads to coordinate scission of mitochondrial membranes^15,17^.

It has been established that androgens, essential for normal Leydig cell development and function, mediate their biological effects through binding to the intracellular androgen receptors (ARs). For instance, in the testicular-feminized (Tfm) mice lacking the AR, Leydig cell number and testicular androgen synthesis are markedly reduced as a consequence of the impairment of the expression of genes required for normal steroidogenic function of Leydig cells^20,21^. Similarly, de Gendt et al*.*^22^ demonstrated that in mice with total *AR* knockout a reduction of Leydig cell numbers was higher than in mice with Sertoli cell-specific knockout of the *AR* gene (SCARKO) what evidences the significance of AR-signaling in Leydig cells per se. It should be pointed out, however, that in SCARKO mice Leydig cells were larger, displayed increased volume of mitochondria and lipid droplets and expressed higher levels of the proteins and transcripts crucial for the formation of steroidogenic enzymes.

Flutamide is the pure non-steroidal AR antagonist that blocks receptor binding of androgen, blocks nuclear retention of the AR complex and slows down its transcriptional activity, disturbing the action of endogenous testosterone^23–25^. Flutamide belongs to a group of anti-androgens with many applications, from basic science to clinics; it is specifically used in the endocrine therapies and androgen-dependent diseases such as prostate cancer and scalp hair loss in men or hirsutism in women (for review see^26,27^). It has been repeatedly shown that compounds with anti-androgenic activity (including flutamide) have the potential to adversely affect human and animal reproduction leading to several disorders of male reproductive system in adulthood^28–30^. Substantial increases in plasma LH and testosterone concentrations shortly after acute treatment with flutamide were reported in studies on male rats and hamsters^31,32^. Several further experiments showed that anti-androgens (and specifically flutamide) disturbing ability of testosterone receptor binding, lead not only to weakening of the negative feedback of testosterone on the HPG axis but also induce impairment of cellular processes controlling spermatogenesis and steroidogenesis in testes, both of humans and animals^33,34^ (for review see^35^). On the other hand, information on a relationship between Leydig cell steroidogenic function and mitochondrial activity at the electron microscopic (EM) level is still limited. In a series of experiments we have demonstrated that AR blockage initiated by neonatal flutamide exposure affects testis morphology, alters the expression of intercellular junction proteins at the blood-testis barrier, and leads to androgen–estrogen imbalance in adult boar testes^36–38^. In subsequent publications we showed that flutamide treatment leads to alterations of the intercellular junction ultrastructure and enlargement of the interstitial tissue of adult rat testis^39,40^. A rationale for performing experiments reported herein comes from our latest study using the same animal model demonstrating altered functioning of adipokines (1), increase in cholesterol availability in Leydig cells (2), sixfold increase in plasma testosterone concentration (3) and up-regulation of the translocator protein (TSPO) gene expression level in testes as a result of short-term flutamide exposure^41^.

Based on the aforementioned data, we previously hypothesized^41^ that flutamide administration (and subsequent blockade of the AR) leads to elevated steroidogenic activity of Leydig cells. In this context, we set out to detect the gene expression levels of proteins required for the first two steps of testosterone synthesis pathway: steroidogenic acute regulatory protein (StAR), involved in transferring cholesterol from the outer mitochondrial membrane to the inner mitochondrial membrane (1), cytochrome P450 cholesterol side-chain cleavage enzyme (CYP11A1) residing on the matrix side of the inner mitochondrial membranes and converting cholesterol to pregnenolone (2), and 3β-hydroxysteroid dehydrogenase (3β-HSD), acting within or outside the mitochondria and involved in sequential conversions of pregnenolone to testosterone (3). Effects of short-term androgen signaling disruption caused by flutamide on (1) intratesticular steroid hormone levels (testosterone and estradiol), (2) the expression of cytochrome P450 aromatase (CYP19A1), acting in the SER and ensuring testosterone conversion to estradiol, and (3) plasma LH content, were also determined. Furthermore, we would like to stress that we performed all above listed analyses for better understanding of alterations in the production of sex hormones observed after flutamide treatment.

To get further insight into the functioning of Leydig cells after flutamide treatment, we investigated the ultrastructure of Leydig cells in untreated (control) and treated rats. As the information that can be extracted from single, often incidental and taken at different angles sections, is not sufficient for the accurate visualization of spatial relationships of the cell constituents, we performed a computer aided 3D reconstruction of serial ultrathin sections of Leydig cells in treated versus untreated rats. These analyses showed that ultrastructure of Leydig cells in treated animals is substantially altered and that observed alterations correspond well to the results of biochemical and molecular tests. This, in turn, confirms the idea that impaired androgen signaling caused by flutamide administration results in activation of the LH axis, enhanced Leydig cell stimulation and consequent rise of their steroidogenic activity.

## Results

### Flutamide-induced alterations in steroidogenic proteins’ gene expression levels

**Figure 1:**
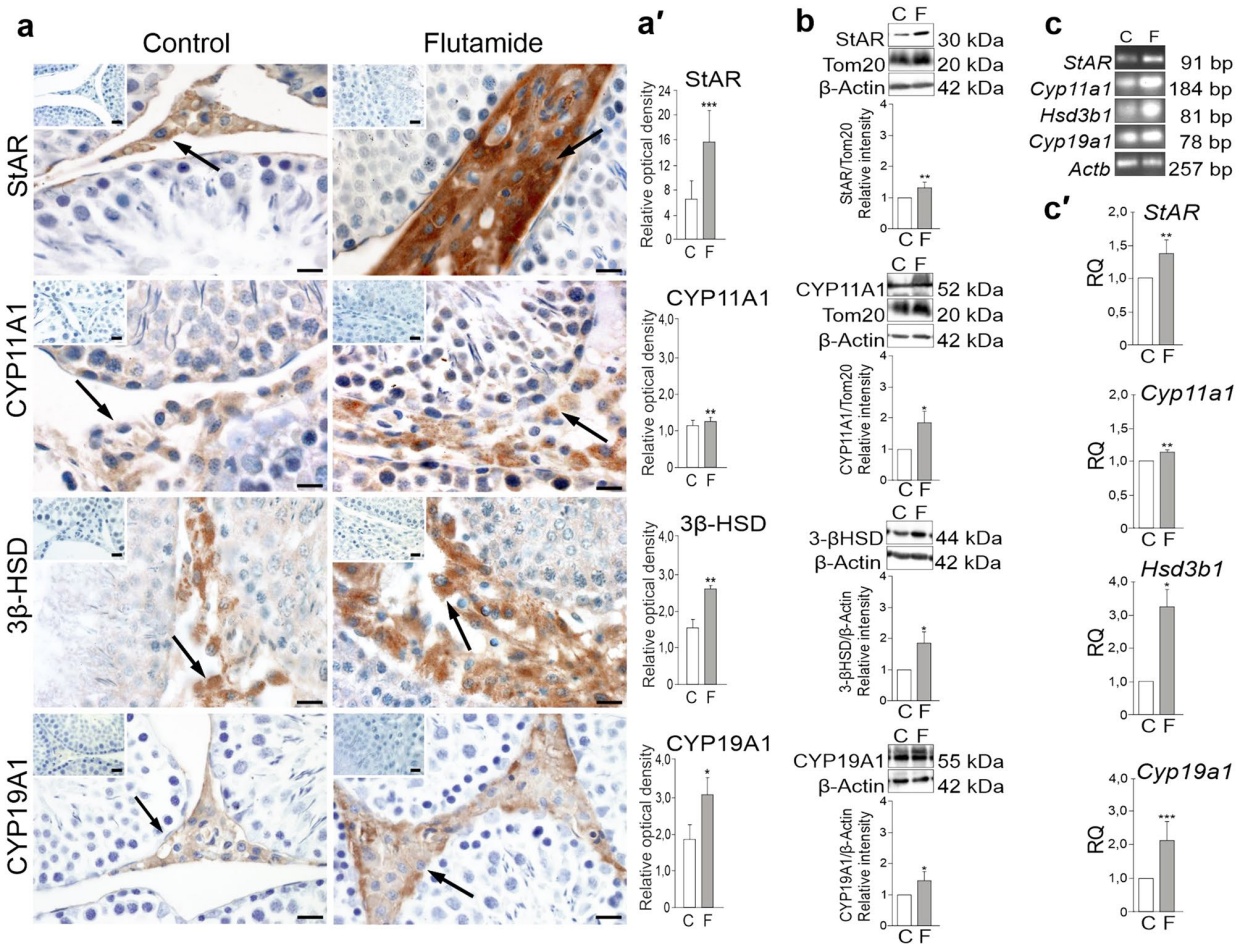
Immunohistochemical localization of StAR, CYP11A1, 3β-HSD, and CYP19A1 (**a)** and quantitative image analyses displayed as relative optical density (**a′**), protein (**b**) and mRNA (**c**,**c′**) expression levels in testes of control and flutamide-treated rats. Counterstaining with Mayer’s haematoxylin. Bars represent 20 μm. (**a**). Representative microphotographs show the positive signal of StAR, CYP11A1, 3β-HSD, and CYP19A1 proteins as localized to Leydig cells (arrows). Note higher intensity of the staining for StAR, CYP11A1, 3β-HSD, and CYP19A1 in Leydig cells of flutamide-treated rats compared to respective controls (**a**,**a′**). No immunopositive staining for StAR, CYP11A1, 3β-HSD, and CYP19A1 proteins are observed when the primary antibodies were omitted (top view inserts in **a**). (**b**) Representative western blots and the relative levels of StAR, CYP11A1, 3β-HSD, and CYP19A1 proteins normalized to β-Actin, which served as an internal protein loading control. The relative levels of StAR and CYP11A1 were normalized to a mitochondrial protein, Tom20, which served as an additional control protein. Protein levels within control testes were given a value of 1. Displayed are the cropped blots and original blots are provided in Supplementary Fig. S6. (**c**,**c′**) Representative RT-PCR and qRT-PCR analyses of *StAR, Cyp11a1, Hsd3b1, and Cyp19a1* mRNA expression levels, respectively. Relative quantification (RQ) is expressed as mean ± SD. Statistically significant differences from control values are denoted as *P < 0.05; **P < 0.01; ***P < 0.001). Control (n = 6) and flutamide-treated (n = 6) animals.

Effects of flutamide on gene expression levels in rat testis were determined using immunohistochemistry, western blotting, and real-time RT-PCR (Fig. 1a–c′). Positive immunostainings for StAR, CYP11A1, 3β-HSD, and CYP19A1 were confined to Leydig cells of both control and flutamide-treated rats. Following flutamide treatment, however, all the stainings were of higher intensity than those of the respective controls (Fig. 1a). The most apparent increase in the staining intensity was observed for StAR protein, what was confirmed by quantitative densitometric image analysis (*P* < 0.001) (Fig. 1a′). Also, qualitative results of CYP11A1, 3β-HSD, and CYP19A1 (Fig. 1a) were confirmed by quantitative analyses (*P* < 0.05; *P* < 0.01) (Fig. 1a′). β-Actin, used as an unrelated control protein, showed unchanged immunoreactivity in flutamide-treated versus control tissues (Supplementary Figure S5). Following western blot analysis, the proteins were observed as single bands near 30 kDa (StAR), 52 kDa (CYP11A1), 44 kDa (3β-HSD), 55 kDa (CYP19A1), 42 kDa (β-Actin), and 20 kDa (Tom20). Significant increases in the levels of StAR, CYP11A1, 3β-HSD, and CYP19A1 proteins were found in testes of flutamide-treated rats (*P* < 0.05; *P* < 0.01) compared to those of controls (Fig. 1b). Of note, the expression of Tom20 (a protein of the outer mitochondrial membrane) evaluated in parallel served as an additional control for the normalization of signals of mitochondrial proteins StAR and CYP11A1. Electrophoresis revealed that PCR products matched with the expected sizes of 91, 184, 81, 78 bp, and 257 bp for *StAR*, *Cyp11a1*, *Hsd3b1*, *Cyp19a1*, and *Actb*, respectively (Fig. 1c). Up-regulation of *StAR*, *Cyp11a1*, *Hsd3b1*, *Cyp19a1* mRNAs were detected in testes of flutamide-treated rats (*P* < 0.05; *P* < 0.01; *P* < 0.001) compared to those of controls (Fig. 1c′).

Alterations in *StAR*,* Cyp11a1*,* Hsd3b1*, and *Cyp19a1* gene expression levels suggest that the exposure to flutamide may increase Leydig cell steroidogenic activity.

### Flutamide-induced alterations in plasma LH concentration and testosterone and estradiol levels in testicular homogenates

Effects of flutamide on LH and steroid hormone levels were determined using ELISA assays (see Supplementary Table S1). LH concentration in blood plasma increased significantly (*P* < 0.01) in flutamide-treated (33.760 ± 2.514 mIU/mL) relative to control-treated samples (24.380 ± 4.048 mIU/mL). Intratesticular testosterone (51.170 ± 2.423 ng/mL) and estradiol (45.927 ± 9.935 pg/mL) levels also significantly increased (*P* < 0.01 and *P* < 0.05, respectively) following flutamide exposure compared to the control values (32.040 ± 3.722 ng/mL and 24.380 ± 3.966 pg/mL), respectively.

Elevated plasma LH content may indicate the presence of flutamide-induced effects within the HPG axis. This may lead to enhanced capacity of Leydig cells to produce and metabolize testosterone, manifested by increase in the intratesticular levels of testosterone and estradiol.

### Flutamide-induced alterations in morphology and ultrastructure of Leydig cells

**Figure 2:**
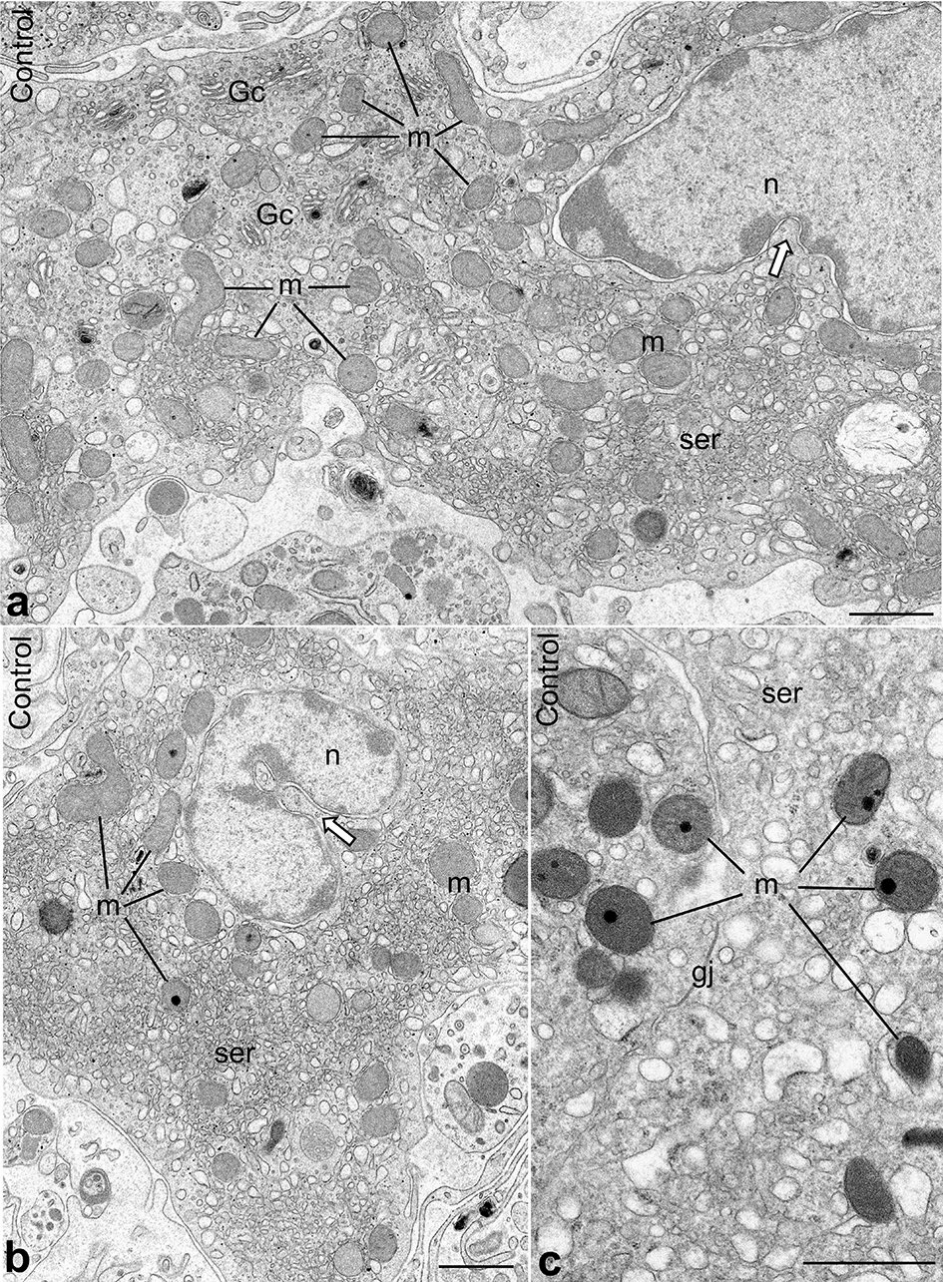
Ultrastructure of Leydig cells from control animals. (**a**,**b**) Random sections through the central (containing nucleus) cell region. Note relatively small, predominantly spherical mitochondria (m), SER elements (ser), Golgi complexes (Gc) and cell nucleus (n). Thick white arrows indicate infoldings of the nuclear envelope. (**c**) Gap junction (gj) between two adjacent Leydig cells. Bars represent 1 µm.

**Figure 3:**
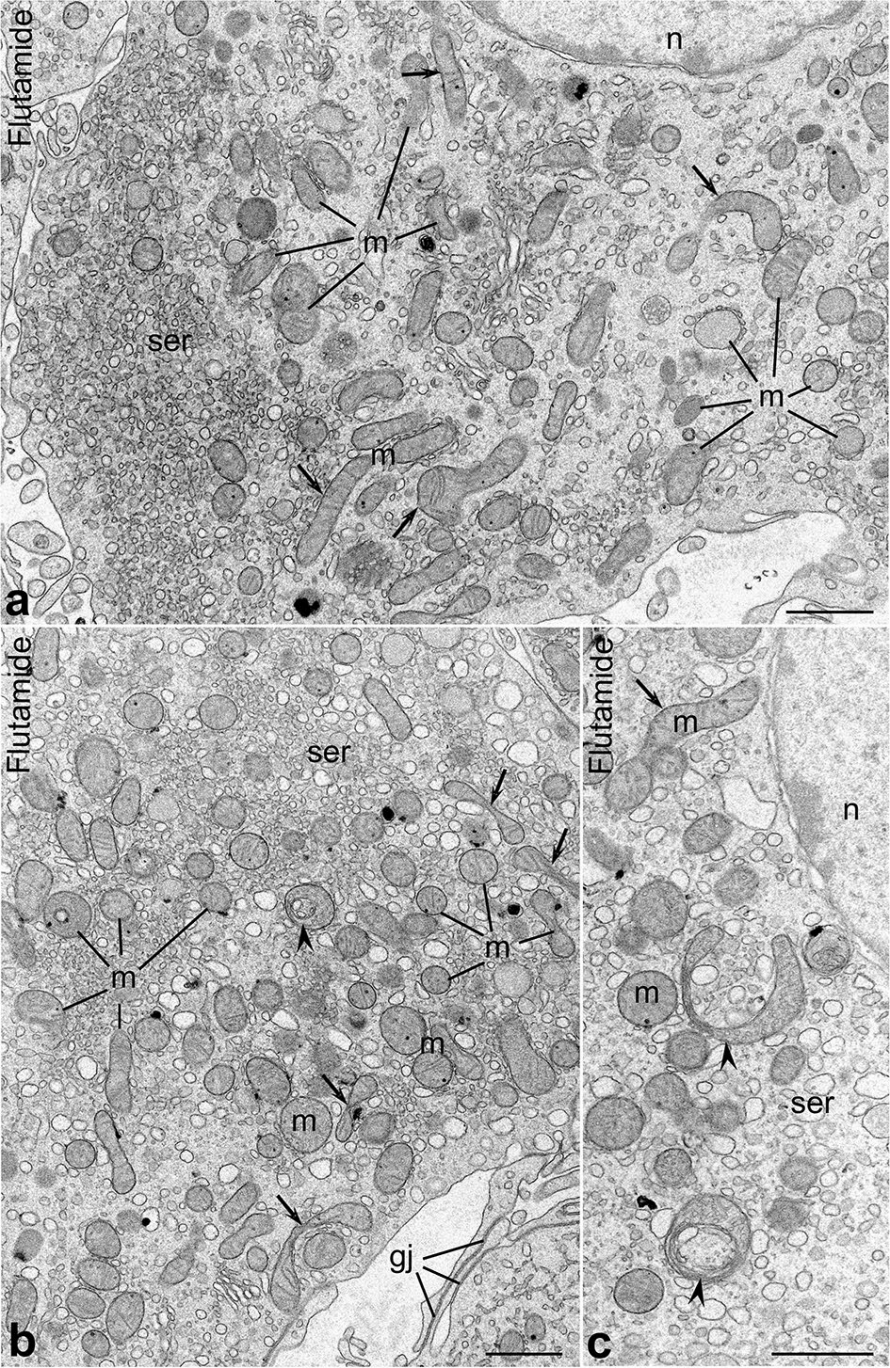
Ultrastructure of Leydig cells from experimental animals. (**a**) Section through a central region of highly altered cell. (**b**) Section through slightly altered cell; note the cytoplasm region overloaded with SER elements. (**c**) Higher magnification of a perinuclear cytoplasm of highly active cell; note morphologically modified mitochondria. Mitochondria (m), SER elements (ser), cell nucleus (n), arrows indicate highly elongated mitochondria, arrowheads—u shaped and “circular” ones. Bars represent 1 µm.

Morphology and ultrastructure of Leydig cells from control and flutamide-treated rats (experimental animals) were analyzed at the level of light (Supplementary Figure S1) and electron microscopy (TEM) (Figs. 2, 3, Supplementary Figure S2–S4).

#### Control rats

Leydig cells in testes of control rats displayed typical morphology (Supplementary Figure S1). Therefore, the following description is presented only for comparison with the next section. As a rule, Leydig cells from control rats were slightly elongated or spindle-shaped. Their nuclei were more or less centrally placed and surrounded with slightly undulating nuclear envelope (Fig. 2, Supplementary Figure S2), however, deep and regular infoldings of the envelope were frequently found (Fig. 2a,b; white thick arrows). The cytoplasm comprised typical organelles: Golgi complexes (predominantly in the neighborhood of the nucleus), vesicles and short cisternae of SER as well as mitochondria (Fig. 2a,b, Supplementary Figure S2). The latter were more or less evenly distributed. Mitochondria had regular shapes; spherical and ovoid forms were apparently predominant, whereas elongated and/or bifurcated ones were seldom. Large, slightly bent gap junctions were often encountered between adjacent Leydig cells (Fig. 2c, gj).

#### Experimental animals

In treated males, the Leydig cells were larger than in the control ones (Supplementary Figure S1, yellow asterisks) as confirmed by morphometric analysis (see the next section). The morphology and ultrastructure of Leydig cells in treated males was also apparently altered, however, the degree of morphological changes observed in individual cells varied substantially. These variances were especially evident in the distribution and morphology of mitochondria (see the next section). In some cells, mitochondria were structurally similar to those of control animals (Fig. 3b, Supplementary Figure S3, S4). These cells were classified as “slightly altered”. In others, the mitochondria were not only much more numerous and tightly clustered, but exhibited several morphological modifications (Fig. 3a,c, Supplementary Figure S3, S4). In such cells, classified as “highly altered”, elongated, u-shaped, swollen and even “circular” (see the next section) mitochondria were often found (Fig. 3, arrows and arrowheads, Supplementary Figure S4). In addition to changes in mitochondrial morphology, we noticed also alterations in the organization of the SER. Namely, in Leydig cells from experimental animals SER elements (vesicles) were not as expanded as those of control animals (compare Figs. 2, 3, Supplementary Figure S2–S4). Additionally, in some highly altered Leydig cells “segregation” of cellular organelles was evident; in such cells certain regions (predominantly peripheral) of cytoplasm overloaded solely with SER elements were clearly separated from regions comprising mitochondria associated with SER (Fig. 3a, Supplementary Figure S4).

In addition to the described alterations in the morphology of cytoplasmic constituents of the Leydig cells, we noticed also modifications in the organization of intercellular junction present between apposed cells. Namely, the number of gap junctions encountered between Leydig cells and/or their projections was higher in treated animals than in the control ones (Fig. 3b, gj). Moreover, careful analysis of appropriately oriented sections clearly indicated that individual gap junctions are not only more numerous but also larger, more extensive (compare Figs. 2, 3gj).

#### Morphometric analyses

Morphometric analysis of Leydig cells from experimental versus control animals was performed on the semithin micrographs. The analysis revealed significantly higher (*P* < 0.001) mean area of Leydig cells (40.628 ± 12.616 μm^2^ vs. 22.260 ± 6.504 μm^2^). Accordingly, the mean number of Leydig cells per 1000 μm^2^ of the interstitial tissue was significantly reduced (*P* < 0.001) in testes of experimental animals (12.972 ± 2.662) compared to the control (22.775 ± 5.405).

Results of morphometric analyses of selected parameters of Leydig cells from control and flutamide treated rats are presented in Table 1. All the measurements were taken on the EM micrographs selected at random. In accordance with above cytological description:

- the percentage of the cell area occupied by mitochondria, mean mitochondria perimeter, and the percentage of morphologically modified mitochondria were significantly higher in Leydig cells (both slightly and highly altered) from rat testes exposed to flutamide (*P* < 0.001) than from those of controls;
- mean mitochondria perimeter, and the percentage of modified mitochondria were significantly higher in highly altered Leydig cells (*P* < 0.05 and *P* < 0.01 respectively) than in slightly altered ones.
- mean diameter of SER vesicles was significantly lower in slightly altered and highly altered cells (*P* < 0.05 and *P* < 0.001 respectively) than in control cells;
- mean diameter of SER vesicles was significantly lower in highly altered cells (*P* < 0.01) than in slightly altered ones.

**Table 1:** Morphometric analysis of selected parameters of Leydig cells from control and flutamide-treated animals.

Analyzed parameters	Control cells	Slightly altered cells	Highly altered cells
Mean mitochondria perimeter (μm)	1.407 ± 0.530	1.866 ± 0.086 ***	2.009 ± 0.099***/^a^
Percentage of cytoplasmic area occupied by mitochondria (%)	5.256 ± 1.784	16.783 ± 2.507***	17.499 ± 1.871***
Percentage of morphologically modified mitochondria (%)	2.750 ± 0.898	6.857 ± 2.752***	15.291 ± 2.223***/^b^
Mean diameter of SER vesicles (μm)	0.286 ± 0.072	0.213 ± 0.043*	0.151 ± 0.025***/^b^

Data are expressed as means ± SD (n = 6 animals/each group). *P < 0.05 versus control cells; ***P < 0.001 versus control cells; ^a^*P* < 0.05 versus slightly altered cells; ^b^*P* < 0.01 versus slightly altered cells.

#### Computer aided reconstruction of Leydig cell morphology

**Figure 4:**
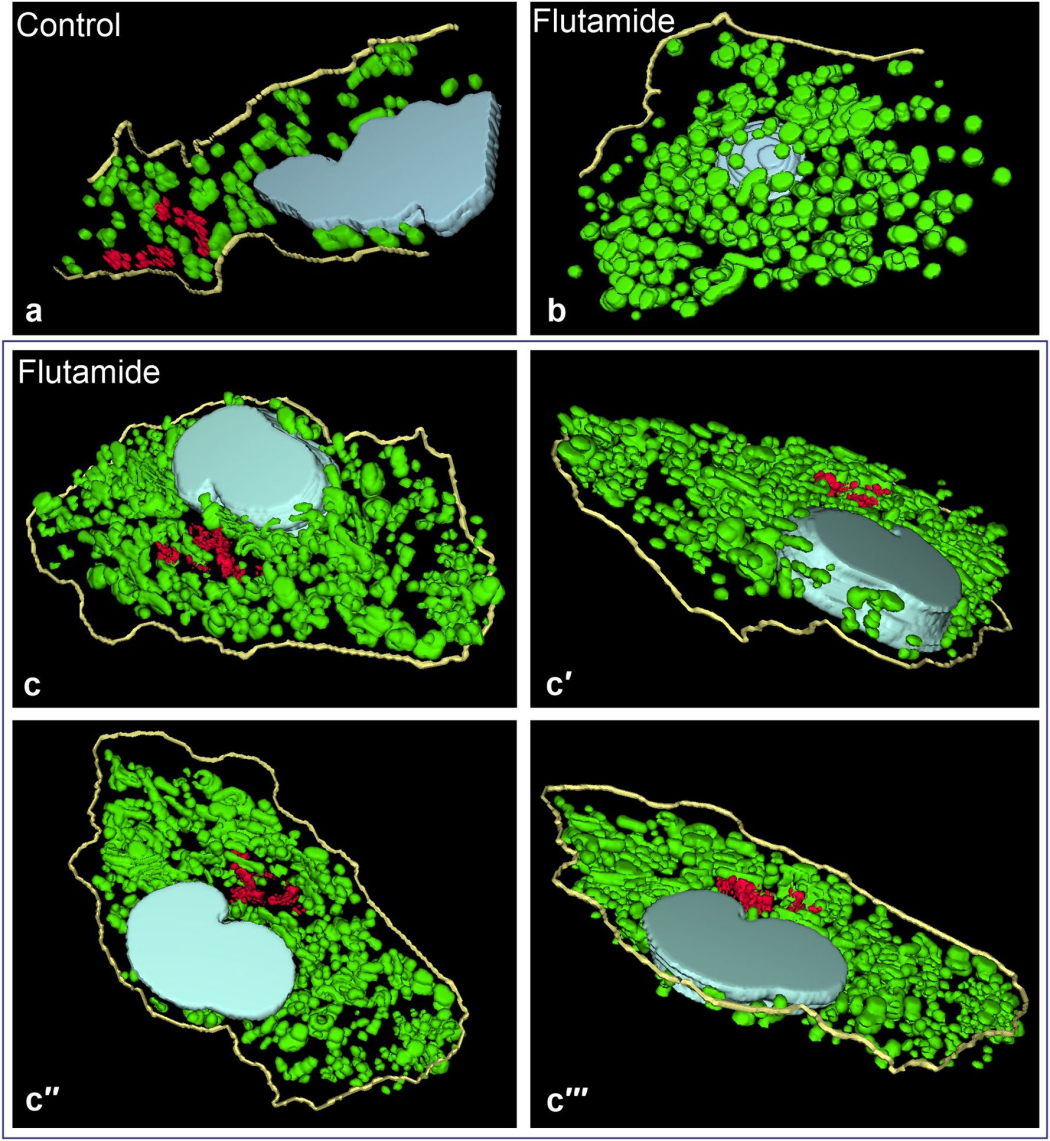
Computer aided 3D reconstructions of Leydig cells. (**a**) A sample 3D reconstruction of a “control” Leydig cell. (**b**,**c**) Sample 3D reconstructions of Leydig cells treated with flutamide. (**b**) A slightly altered cell. (**c**-**c‴**) A highly altered cell as seen from 4 different angles. Note mitochondria/mitochondrial networks (green), Golgi complexes (red), cell nuclei (light blue) and the cell outline (yellow). Three-dimensional reconstructions images were performed with the ImageJ Software (version 1.51 h), https://imagej.nih.gov/ij/.

**Figure 5:**
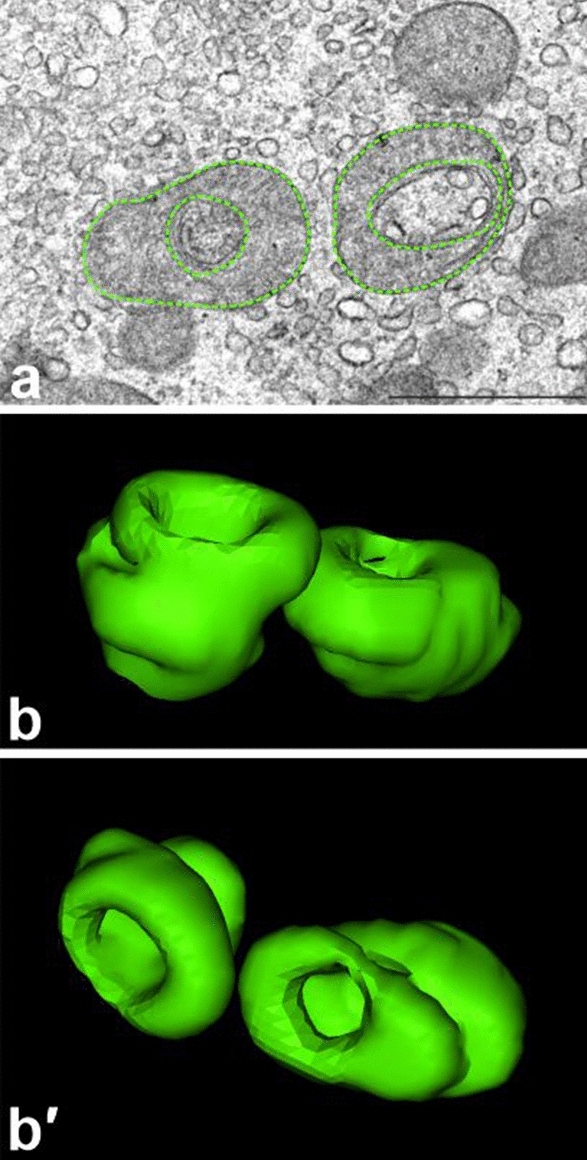
Goblet-shaped mitochondria. (**a**) Transverse section through two “circular” mitochondria; their outlines (external and internal) are marked with green dots. Bar represents 2 µm. (**b**,**b′**) 3D reconstruction of the same mitochondria as seen from two different angles; “circular” organelles shown in (**a**) appeared to represent cross sections of goblet-shaped mitochondria. Three-dimensional reconstructions images were performed with the ImageJ Software (version 1.51 h), https://imagej.nih.gov/ij/.

For exact visualization of mutual relationships between cell organelles at the EM level, we performed a computer aided 3D reconstruction of serial ultrathin sections. Between 9 and 15 ultrathin sections were used in our reconstructions. Analysis of the obtained 3D images (Fig. 4; sample animated reconstructions are presented in Supplementary Videos S1–S4) have confirmed that morphology of Leydig cell from treated animals is distinctly altered and that degree of observed morphological changes varies in individual cells (compare Fig. 4b,c-c‴). In some Leydig cells (termed above “slightly altered”) mitochondria, even though more numerous than in Leydig cells of control animals, were relatively small and more or less spherical (compare Fig. 4a,b). In other cells (termed “highly altered”) mitochondria are not only more numerous but also interconnected forming local networks (Fig. 4c-c‴). Careful analysis of 3D reconstructions of highly altered cells showed additionally that “circular mitochondria” often observed during conventional EM analysis, represent in fact cross sections of specific cup- or goblet-shaped mitochondria (Fig. 5). At the present stage of analysis, we do not know whether such shape of mitochondria is related to a specific physiological state or not.

### Flutamide-induced alterations in Drp1 and Mfn2 gene expression

**Figure 6:**
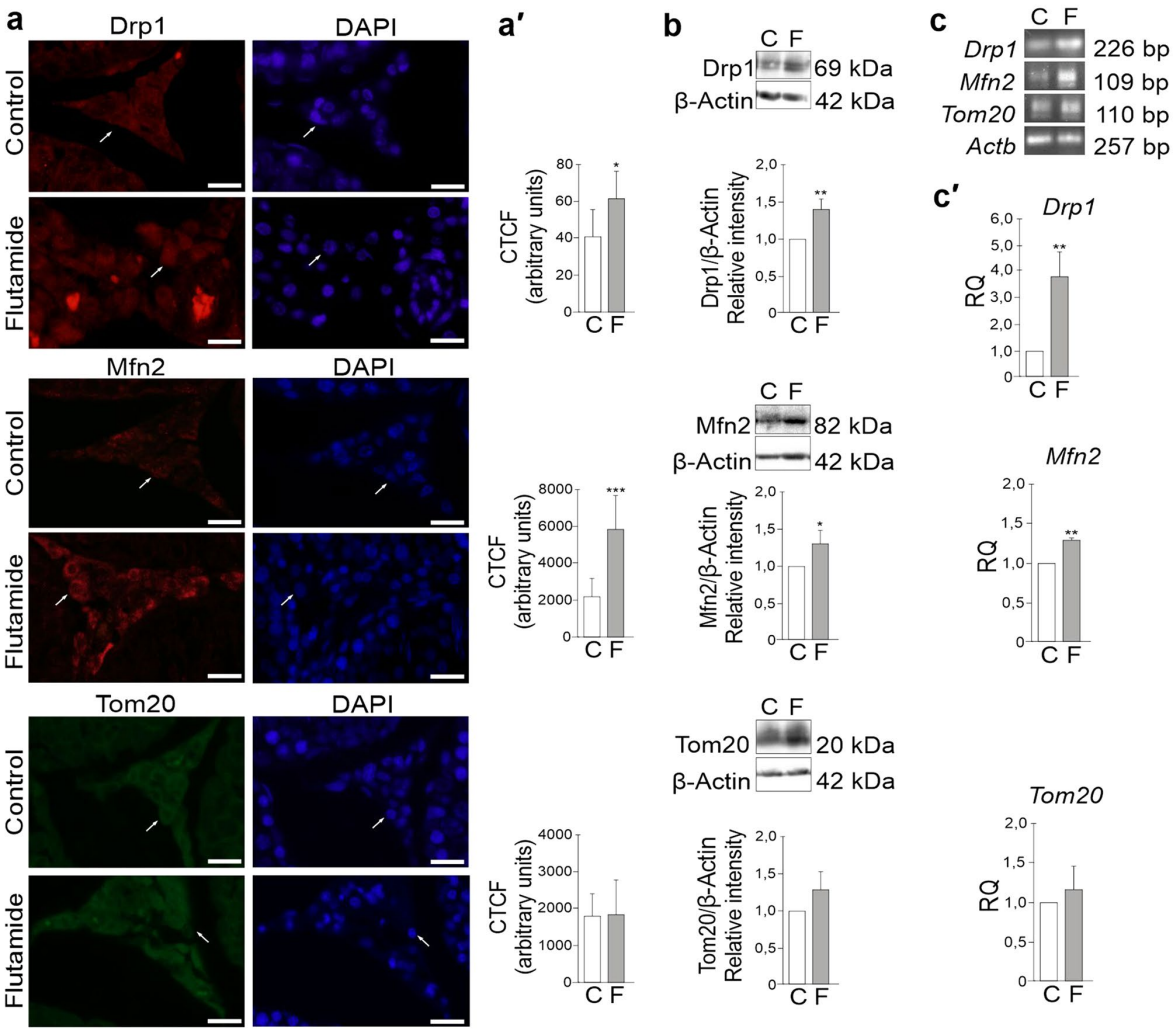
Immunofluorescence localization of Drp1, Mfn2, and Tom20 (control protein) (**a**), quantitative image analysis displayed in corrected total cell fluorescence (CTCF) (**a′**)**,** Drp1, Mfn2, and Tom20 protein levels (**b**) and *Drp1, Mfn2,* and *Tom20* mRNA (**c**,**c′**) expression levels in testes of control and flutamide-treated rats. Bars represent 20 μm. (**a**) Representative microphotographs show positive signals for Drp1 and Mfn2 (red) and for Tom20 (green) localized to Leydig cells (arrows). Cell nuclei were stained with DAPI (blue) (arrows). Note higher signal intensity of Drp1 and Mfn2 but not of Tom20 in Leydig cells of flutamide-treated rats compared to controls (**a′**). (**b**) Representative western blots and the relative levels of Drp1, Mfn2, and Tom20 proteins normalized to β-Actin, which served as an internal protein loading control. Drp1, Mfn2, and Tom20 protein levels within control testes were given a value of 1. Displayed are the cropped blots and original blots are provided in Supplementary Fig. S6. (**c**,**c′**) Representative RT-PCR and qRT-PCR analyses of *Drp1, Mfn2,* and *Tom20* expression levels, respectively. Relative quantification (RQ) is expressed as mean ± SD. Note significant increase in Drp1 and Mfn2 protein and mRNA levels and slight (not statistically significant) increase in Tom20 protein and mRNA levels in testes homogenates of treated rats compared to controls. Statistically significant differences from control values are denoted as *P < 0.05; **P < 0.01. Control (n = 6) and flutamide-treated (n = 6) animals.

As in Leydig cells from treated animals, the mitochondria are not only more numerous (as compared to the Leydig cells from control rats) but also fuse and form extensive networks, we decided to test whether these morphological alterations involve mitochondrial fission/multiplication and fusion—key processes participating in mitochondrial homeostasis (see “Introduction” section). Therefore, we stained paraplast sections with antibodies against two proteins mediating mentioned processes in vertebrates: dynamin-related protein 1 (Drp1), a major regulator of mitochondrial fission^39,40^, and mitofusin, Mfn2 participating in mitochondrial fusion^53^. Immunofluorescence analysis revealed positive signals for both proteins (Drp1 and Mfn2) throughout the interstitial area of control and flutamide-treated rats (Fig. 6a). The signals were localized to Leydig cells and its intensity increased following flutamide exposure compared to the control (*P* < 0.05 and *P* < 0.001, respectively) as confirmed by quantitative image analysis (Fig. 6a′). In accordance to the results of immunofluorescence analyses, significant increases in levels of Drp1 and Mfn2 proteins (*P* < 0.01; *P* < 0.05) (Fig. 6b) and up-regulation of *Drp1* and *Mfn2* mRNAs (*P* < 0.01; *P* < 0.01) were detected in testes of flutamide-treated rats compared to those of controls (Fig. 6c′). No changes in immunofluorescence and a minor, not statistically significant increase in Tom20 protein and mRNA levels were noticed following flutamide compared to the controls (Fig. 6a,a′,b,c,c′). Proteins (detected by western blot) were observed as single bands near 69 kDa (Drp1), 82 kDa (Mfn2), 20 kDa (Tom20) and 42 kDa (β-Actin) (Fig. 6b), while electrophoresis revealed PCR-amplified products of the predicted sizes; 226 bp for *Drp1*, 109 bp for *Mfn2,* 110 bp for *Tom20,* and 257 bp for *Actb* (Fig. 6c).

Our findings suggest that multiplication of mitochondria and consecutive formation of extensive mitochondrial networks observed in Leydig cells after flutamide treatment, being an obvious result of enhanced biogenesis of these organelles might involve also proteins regulating mitochondrial dynamics, e. g. Drp1 and Mfn2.

## Discussion

Although biosynthesis of testosterone in Leydig cells and the regulation of this process are well established and have been discussed in several reviews^4,5,42,43^, the relationship between the disruption of androgen signaling (caused by exposure to flutamide) and consequent enhancement of the expression of proteins involved in steroid hormone conversions on one hand, and the ultrastructure of Leydig cell constituents, including mitochondria, on the other, is not fully understood.

In the present paper we demonstrated that immunoexpression of three crucial steroidogenic proteins, namely the StAR, CYP11A1 and 3β-HSD was restricted to Leydig cells, in control as well treated (with flutamide) rats. We showed also that in our model the staining intensity of all the three proteins was quantitatively higher in treated versus control animals. These results are in line with our biochemical and molecular analyses showing increased level of the StAR, CYP11A1 and 3β-HSD as well as distinct up-regulation of their mRNAs in testicular homogenates of flutamide-treated rats as compared to controls. Notably, flutamide-induced changes in the steroidogenic activity correspond well with elevated intratesticular testosterone and estradiol concentrations (compare Fig. 1 and Supplementary Table S1). Similar results have previously been reported by Ohsako et al.^33^ and Sarrabay et al.^34^. In the first paper elevated levels of testosterone and steroidogenic enzymes in rat testes exposed to flutamide at a dose 25 mg/kg bw/day for 6 days were reported; in the second up-regulation of the expression of steroidogenic enzymes in testicular tissue as well as increase of plasma testosterone concentration after administration of flutamide (1–10 mg/kg bw/day) during 28 days were documented. As in our study short (seven day) exposure to higher concentration of flutamide (50 mg/kg bw/day) also induced distinct changes in the steroidogenic proteins’ expression and the hormone levels, it seems likely that shorter time of exposure to flutamide can be compensated by its higher dose. Indeed, in our latest report using the same dose and duration of flutamide exposure as reported in the present study, a significant increase in plasma testosterone content in adult rats was demonstrated^41^. All above results together with elevated plasma LH concentration (documented in the present study) support earlier findings showing that flutamide leads to the inhibition of the negative feedback of androgens in the HPG axis (by preventing androgen binding to the AR at the level of hypothalamus^44–46^ and to the enhancement of Leydig cell steroidogenic activity (e.g.^34^). Notably, besides regulating HPG axis, androgens are involved also in autocrine control of Leydig cell steroidogenic enzymes^47^. This notion is in line with in vitro studies showing that flutamide acting directly on Leydig cells, affects the expression of steroidogenic proteins^48^.

As we found that the level of intratesticular estradiol was substantially elevated in treated rats versus controls, we have decided to examine the expression of aromatase (CYP19A1), that converts testosterone to estradiol. As expected, increased mRNA and protein expression levels of CYP19A1 were detected in testes of experimental animals, indicating enhanced testosterone metabolism after flutamide exposure. In contrast, Ohsako and co-workers^33^, using different methodology of gene expression analysis, did not reveal any changes in aromatase expression upon flutamide treatment. In earlier study, however, Reznikov et al.^49^ reported alteration of testicular metabolism of testosterone in guinea pigs treated with an anti-androgen, 4-nitro-3-trifluoromethylisobutyranilide (NFBA).

In addition to flutamide-induced changes in levels of steroidogenic proteins, their mRNAs and hormones (testosterone, estradiol), we showed also that the testicular interstitial area after flutamide treatment is apparently enlarged (for comparison, see flutamide vs. control images in Fig. 1a) and contains hypertrophic Leydig cells. This observation is in line with the earlier publications^49,50^ reporting enlarged Leydig cells of guinea pigs following NFBA or flutamide administration. It is worth adding here that similar effect of flutamide exposure was noticed in rats—more than 40 years ago^51^. Moreover, an apparent enlargement of the interstitial area containing hypertrophic Leydig cells was also observed in testes of mature boar injected with flutamide neonatally^36^ and in transgenic mice overexpressing aromatase (AROM^+^)^52^. It is to the point to add here that O’Hara and coworkers^47^ demonstrated in a series of experiments that selective ablation of AR in adult Leydig cells leads to hypertrophy of Leydig cells and increase in estrogen signaling. Interestingly, the same study revealed no changes in aromatase expression, consequently suggesting that the expression of this enzyme is not controlled by Leydig cell AR. In the light of this results, up-regulation of aromatase in our model seems to result from increased stimulation of Leydig cells with LH, rather than from direct blockade of Leydig cell AR by flutamide. Similar stimulatory effect of LH on aromatase expression in Leydig cells was also documented by several earlier studies^53–55^. It is tempting to speculate, in the context of above data that enlargement of the testicular interstitium and Leydig cell hypertrophy as observed in this study after flutamide treatment is associated with enhanced expression of aromatase (CYP19A1) and consequent elevated estradiol level in testes.

As increased expression of steroidogenic proteins and a higher concentration of testosterone in testes of experimental animals might involve higher activity of Leydig cell mitochondria, we have decided to compare morphology and ultrastructure of Leydig cells in control versus experimental animals. The EM analysis, showed that the Leydig cell mitochondria in treated rats were more numerous (1), morphologically modified (2) and often tightly clustered (3). As the degree of above morphological alterations observed in individual cells varied substantially, we classified cell from treated animals into two categories termed “slightly” and “highly” altered. Performed 3D reconstructions of serial ultrathin sections revealed that after flutamide treatment mitochondria are not only more numerous but fuse forming local mitochondrial networks (see Supplementary Videos S1–S4). This effect was especially evident in highly altered Leydig cells (see Fig. 4c-c‴ and Supplementary Videos S3, S4). The comparison of above morphological data on one side and the results of morphometric analyses (see Table 1) on the other, leads to the conclusion that the total volume of the Leydig cell mitochondria in treated animals apparently increases, presumably due to enhanced biogenesis of these organelles. The same data indicate additionally that the slightly altered and highly altered Leydig cells do not significantly differ in the number/ volume of their mitochondria. The differences between these two types of cells include degree of fusion of individual mitochondria into a network/s and percentage of morphologically modified mitochondria. Both parameters are apparently lower in slightly altered Leydig cells. It should be stressed here that mitochondrial networks, as a rule consist of mitochondria with higher membrane potential, and therefore highly active (see^14–16^ for further details). In the light of this, we postulate that enhanced steroidogenic activity of Leydig cells from experimental animals is correlated with multiplication of Leydig cell mitochondria and subsequent fusion of individual mitochondria, i.e. formation of active mitochondrial networks. This idea is in line with previous results of the Hales group showing a relationship between Leydig cell steroidogenesis and mitochondrial functionality in MA-10 Leydig tumor cell line^9,11^. On this ground, Hales and coworkers postulated that to support Leydig cell steroidogenesis, mitochondria should be ”energized, polarized, and actively respiring”. Furthermore, enlarged mitochondria were also observed in Leydig cells of AROM^+^ mice showing elevated estradiol concentration^56^. Interestingly, in Leydig cells of adult guinea pig after flutamide treatment, in addition to enlarged volume occupied by organelles required for testosterone biosynthesis (mitochondria, SER), characteristic accumulations of dense osmophilic structures were observed^50^. Morphologically similar electron-dense structures were described in Leydig cells of men treated for more than four years with anti-androgen, bicalutamide^57^. In our EM sections such dense structures were never observed. We believe that this “absence” might be related to different composition of fixatives used during our EM procedure.

Finally, we tested whether multiplication of mitochondria and formation of mitochondrial networks observed in Leydig cells after flutamide treatment involve proteins mediating mitochondrial dynamics. Our tests clearly showed that both protein and mRNA levels of two key regulators of mitochondrial dynamics, namely Drp1 and Mfn2 are significantly increased in Leydig cells of experimental animals as compared to the control ones. It is tempting to speculate, in the light of these results that downstream effects of flutamide treatment, in addition to all other mentioned above effects, comprise also up-regulation of *Drp1* and *Mfn2* transcription.

It should be underlined that results of discussed above EM analyses are obviously consistent with our immunohistochemical, biochemical and molecular approaches. The increased levels of the steroidogenic proteins, up-regulation of their mRNAs and quantitatively higher immunoreactivity (especially the StAR protein, residing in the outer mitochondrial membrane and ensuring cholesterol transfer to the inner mitochondrial membrane), correspond well to higher number of mitochondria, their multiplication and formation of fused, highly active mitochondrial networks. This notion lends an additional support from (1) recent studies reporting that the mitochondrial fusion is required for the correct localization of the StAR protein and expression of its mRNA in MA-10 tumor Leydig cells after hormonal stimulation^58,59^ as well as (2) experimental studies showing importance of highly active (“energized”) mitochondria for proper functioning of Leydig cells in mice^60^.

In addition to the discussed above alterations in the ultrastructure of the Leydig cell cytoplasm, our EM analyses revealed that the number and size of gap junctions connecting neighboring Leydig cells and/or their processes apparently increased after flutamide treatment. One possible explanation for this finding is that gap junctions’ formation is triggered by higher estradiol level in testes following flutamide treatment. Indeed, enhanced expression of connexin43 (the most prominent gap junction protein in testes, Cx43; see^61^ for further details) in Leydig cells, as well as higher estradiol levels in testes of adult rat and boar were demonstrated after short- or long-term flutamide administration^36,40^. Increased estradiol content and increased Cx43 expression level, reported in the human prostate epithelial cells as a consequence of flutamide treatment, additionally support our hypothesis^62^.

## Conclusions

In the study presented herein, we showed that in flutamide-treated rats, distinct increase in the expression level of important steroidogenic proteins (and respective mRNAs) in the testicular tissue as well as elevation of sex steroid hormone (testosterone and estradiol) levels, correspond well to morphological alterations of the Leydig cell ultrastructure. Most importantly, up-regulation of steroidogenic proteins’ expression apparently correlates with multiplication of Leydig cell mitochondria and subsequent formation of local mitochondrial networks in flutamide treated rats. These results suggest that flutamide administration, in addition to previously described effects, enhances the biogenesis of mitochondria and expression of key regulators of mitochondrial dynamics, proteins Drp1 and Mfn2.

## Materials and methods

### Animals and treatment

Adult Wistar male rats were randomly assigned into experimental and control groups (n = 6/each group). Rats from the experimental group were injected subcutaneously with flutamide (Sigma–Aldrich) suspended in corn oil for seven consecutive days (each dose, 50 mg/kg bw), as described previously^39–41^. The selected dose of flutamide was high enough to exert primary effects on cellular targets within the testis without generating testicular germ cell loss from the seminiferous tubules (for further details see^40,41^). The animals were maintained under identical conditions of 12 h light:12 h dark with access to food and fresh water ad libitum. Animals were sacrificed by inhalation with 5% (v/v) isoflurane at 90 days of age. Testes were dissected and blood samples were collected. Plasma were separated from blood samples and used for ELISA analyses. Fresh tissue fragments were either snap-frozen and stored at − 80 °C for qRT-PCR, western blot and ultrastructure examination, or fixed in 4% paraformaldehyde, and embedded in paraplast for immunohistochemistry and immunofluorescence^63^.

The experiments were performed in accordance with Polish legal requirements and in compliance with the Directive 2010/63/EU on the Protection of Animals Used For Scientific Purposes. The use of animals was approved by the First Local Ethical Committee on Animal Testing at the Jagiellonian University in Krakow (permission number: 116/2012) and the 2nd Local Institutional Animal Care and Use Committee in Krakow, Poland (permission number 189/2018). This study was carried out in compliance with the ARRIVE guidelines^64^.

### Immunohistochemistry

For immunohistochemistry 5-micron-thick sections of testes were deparaffinized and rehydrated. Antigen retrieval, endogenous peroxidase activity and non-specific binding sites were blocked as described previously^40^. The sections were incubated overnight at 4 °C with primary antibodies (for details see Supplementary Table S2). On the next day, biotinylated secondary antibody goat anti-rabbit or horse anti-mouse (1:400; Vector Laboratories) was applied. The staining signal was visualized by the avidin-biotinylated horseradish peroxidase complex (1:100; Vectastain Elite ABC Reagent, Vector) and by 0.05% 3.3′-diaminobenzidine tetrachloride dissolved in TBS containing 0.01% (v/v) H_2_O_2_ and 0.07% (wt/v) imidazole. Subsequently, the sections were counterstained with Mayer’s hematoxylin (Vector), dehydrated and coverslipped with DPX mounting medium (Sigma–Aldrich). Control sections included omission of the respective primary antibody and/or substitution by irrelevant IgG.

β-Actin served as unrelated control protein. All procedures were performed identically, at the same time to ensure uniformity and specificity of the staining. Sections were examined with a Leica DMR microscope (Leica Microsystems GmBH).

Images of testicular sections obtained with a digital camera system coupled to an optical microscope (Microphot, Nikon) were used for quantitative analysis by ImageJ Software (NIH). The intensities of IHC staining were calculated as relative optical density (ROD) of reaction products^65^.

### Immunofluorescence

For immunofluorescence labeling testes sections were used. Non-specific binding sites were blocked with normal goat/horse serum. Background fluorescence was reduced by 0.1% solution of sodium borohydride (Sigma-Aldrich). Thereafter, the sections were incubated overnight at 4 °C with anti-Drp1, Mfn2, and Tom20 antibodies (Supplementary Table S2). On the next day, a Cy3-cojugated goat anti-rabbit IgG (1:200; Thermo-Fischer) or Alexa Fluor 488 goat anti-mouse (1:200; Invitrogen) was applied for 60 min as described previously^66^. Lastly, sections were coverslipped with Vectashield mounting medium (Vector) with DAPI and examined with the Leica DMR epifluorescence microscope (FM) equipped with appropriate filters. Mitochondrial protein, positive for the outer membrane, Tom20, served as a control protein, as described previously^67^. For negative control, primary antibody was omitted and no fluorescence was observed.

The fluorescent images were analyzed using ImageJ Software. Single Leydig cell was manually outlined and the area, integrated density and mean gray value were measured. The same outlining procedure was applied to a background zone. The corrected total cell fluorescence (CTCF) has been calculated using following equations CTCF = area of selected cell × mean fluorescence of background readings, as described previously^68^.

### Western blotting

For protein extraction tissue samples were homogenized with cold-ice RIPA buffer (Thermo-Fischer) supplemented with protease inhibitors (Sigma-Aldrich), as described previously^37^. Briefly, tissue lysates were separated by SDS-PAGE, under reducing conditions and transferred to polyvinylidene difluoride membranes (Sigma-Aldrich) through semi-dry transfer (Bio-Rad Laboratories). To prevent nonspecific binding of antibodies, membranes were blocked with a solution of 5% (wt/v) non-fat dry milk containing 0.1% (v/v) Tween 20 and cut at appropriate molecular weights of the prestained molecular weight marker (Thermo-Fischer) to allow the use of different antibodies on different parts of the blot. Next, the blots were probed with the respective primary antibodies (Supplementary Table S2) at 4 °C overnight. Next, secondary antibody conjugated with the horseradish-peroxidase labeled goat anti-mouse or goat anti-rabbit IgG was added (1:3000, Vector) for 60 min at room temperature^40^. The immunocomplexes were detected by chemiluminescence captured with a ChemiDoc XRS + System (Bio-Rad). All blots were stripped and reprobed with an antibody against β-Actin, which was used as a loading control. Mitochondrial protein, Tom20 served as an additional control for the normalization of signals of mitochondrial proteins StAR and CYP11A1. The molecular weights of target proteins were estimated by reference to protein molecular weight marker (Thermo-Fischer). The bands were analyzed and quantified with ImageLab software (Bio-Rad).

### RNA isolation, reverse transcription and real-time quantitative RT-PCR

Total RNA was isolated from the testes with TRIzol reagent (Life Technologies)^66,69^. To minimize DNA contamination TURBO DNase free Kit (Ambion) was used, according to the manufacturer’s protocols. The yield and purity of the collected RNA were evaluated by determining the A260:A280 ratio (NanoDrop ND2000 Spectrophotometer (Thermo-Fischer) and by electrophoresis^37,41^. Only RNA with A260:280 ratios higher than 1.9 was used for cDNA synthesis^66^. RNA was transcribed into cDNA using High-Capacity cDNA Reverse Transcription Kit (Applied Biosystems) according to the manufacturer’s instructions. Parallel reactions for each RNA sample were run in the absence of RT to assess genomic DNA contamination^41^. RNase-free water was added in place of the RT product.

Real-time RT-PCR analyses was carried out using StepOne Real-Time PCR system (Applied Biosystems) as described previously^66,69^. Specific primer pairs (Institute of Biochemistry and Biophysics, Polish Academy of Sciences) were detailed in Supplementary Table S3. Amplification efficiency was calculated as described by Svec et al.^70^ and displayed between 94 and 104%. The specificity and purity of amplification was tested at the end of the PCR by melting curve analysis, and subsequent agarose gel electrophoresis. In qRT-PCR analyses, a negative control corresponding to RT reaction without the reverse transcriptase enzyme and a blank sample were carried out. All experiments were performed in triplicate and repeated in independent experiments three times. mRNA expressions were normalized to the mean expression of reference genes *Rpl13a*, *Actb,* and *Gapdh* mRNA (relative quantification, RQ = 1) with the use of the 2^−ΔΔCt^ method^71^. In Figs. 1 and 6, as an internal control, representative *Actb* transcript level was shown.

### ELISA analysis

Commercially available ELISA kits (DRG International) were used to quantify total testosterone (Cat. No. EIA-1559) and estradiol (Cat. No. EIA-2693) concentrations in testicular homogenates. Additionally, the concentration of LH in plasma was measured (cat.no.: EIA-1289). For all measurements, samples of control and flutamide-treated rats were used according to manufacturer’s protocols. Samples were run in triplicate within the same experiment and measured using a microplate reader (Labtech LT-4500).

### Light and electron microscopy

Dissected testes from both groups were cut into small pieces and immersed in ice-cold pre-fixative containing 2% formaldehyde and 2.5% glutaraldehyde in 0.1 M phosphate buffer, pH 7.3 as described previously^40,63^. Next, fragments of the tissues were washed in the same buffer and post-fixed in a mixture of 2% osmium tetroxide and 0.8% potassium ferrocyanide in 0.1 M phosphate buffer, pH 7.3 for 30 min (4 °C)^40,63^. After dehydration in the graded series of ethanol and acetone the material was infiltrated in a freshly prepared mixture of acetone and epoxy resin (Epoxy Embedding Medium kit, Sigma-Aldrich) and embedded in epoxy resin. Semithin sections (0.7–1 μm thick) were stained with 1% methylene blue in 1% borax and examined under a Leica DMR light microscope^40,63^. Ultrathin sections (80 nm thick) were contrasted with uranyl acetate and lead citrate and analyzed with a JEOL 2100 HT (Japan) TEM^40,63^. Both, semithin and ultrathin sections were used for morphometric analyses of Leydig cells and mitochondria, respectively.

The area of all Leydig cells on each micrograph was measured as a pixel value, using a freehand selection tool in the ImageJ window. Measured pixel values were converted to μm according to the measured pixel value of the scale bar on the corresponding micrographs. The Leydig cells were counted and presented as a mean number of cells per 1000 μm^2^ of the interstitial tissue. Over 36 measurements were made for each animal from control and flutamide-treated animals.

The cell area occupied by mitochondria, mitochondria perimeter, the percentage of morphologically modified mitochondria, and diameter of SER vesicles on each electron micrograph were measured using ImageJ Software (as described above). For each cell, the area occupied by mitochondria was compared to the total cell area and presented as a percentage value. At least six electron micrographs from 6 animals per group were analyzed.

### 3D organization of Leydig cells and morphometric analyses of TEM images

Three-dimensional reconstructions and morphometric analyses of TEM images were performed with the ImageJ Software version 1.51 h^72^. To reconstruct the 3D organization of Leydig cells, serial ultrathin sections of testes were used. Micrographs were aligned to form virtual stack and selected structures (mitochondria, nucleus, Golgi apparatus, cell membrane) were contoured using TrakEM2 plugin. For 3D visualization 3D viewer and Z-projection plugins were used.

### Statistics

The raw data were processed using Statistica 10 software (StatSoft Inc.). Shapiro–Wilk W-test was used to test normality of data distribution and Levene’s test to assessed homogeneity of variance as described previously^41^. Statistical differences in protein and mRNA expression levels were determined using the non-parametric Mann–Whitney U-test. Data were presented as mean ± SD and considered statistically significant at *P < 0.05; **P < 0.01; ***P < 0.001.

## Supplementary information

Supplementary Information.

Supplementary Video S1.

Supplementary Video S2.

Supplementary Video S3.

Supplementary Video S4.
