# Supplementary material for: Flutamide treatment reveals a relationship between steroidogenic activity of Leydig cells and ultrastructure of their mitochondria
Source: Sci Rep. 2021 Jul 2;11:13772. doi: 10.1038/s41598-021-93292-8 (PMC8253797; doi:10.1038/s41598-021-93292-8)
Supplement: Supplementary file 1 — Supplementary Information. [file 41598_2021_93292_MOESM1_ESM.ppt]

## Slide 1
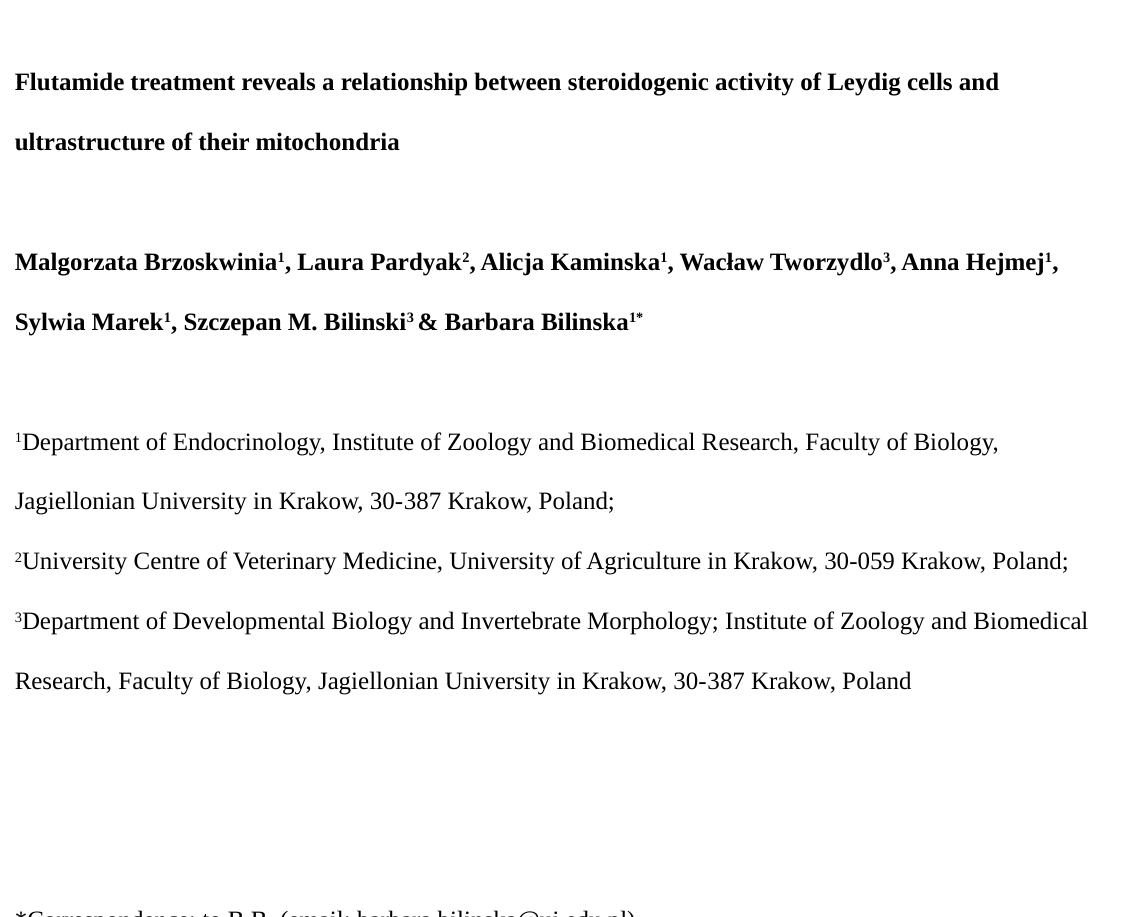

# Flutamide treatment reveals a relationship between steroidogenic activity of Leydig cells and ultrastructure of their mitochondria Malgorzata Brzoskwinia1, Laura Pardyak2, Alicja Kaminska1, Wacław Tworzydlo3, Anna Hejmej1, Sylwia Marek1, Szczepan M. Bilinski3 & Barbara Bilinska1* 1Department of Endocrinology, Institute of Zoology and Biomedical Research, Faculty of Biology, Jagiellonian University in Krakow, 30-387 Krakow, Poland; 2University Centre of Veterinary Medicine, University of Agriculture in Krakow, 30-059 Krakow, Poland; 3Department of Developmental Biology and Invertebrate Morphology; Institute of Zoology and Biomedical Research, Faculty of Biology, Jagiellonian University in Krakow, 30-387 Krakow, Poland   *Correspondence: to B.B. (email: barbara.bilinska@uj.edu.pl)

## Slide 2
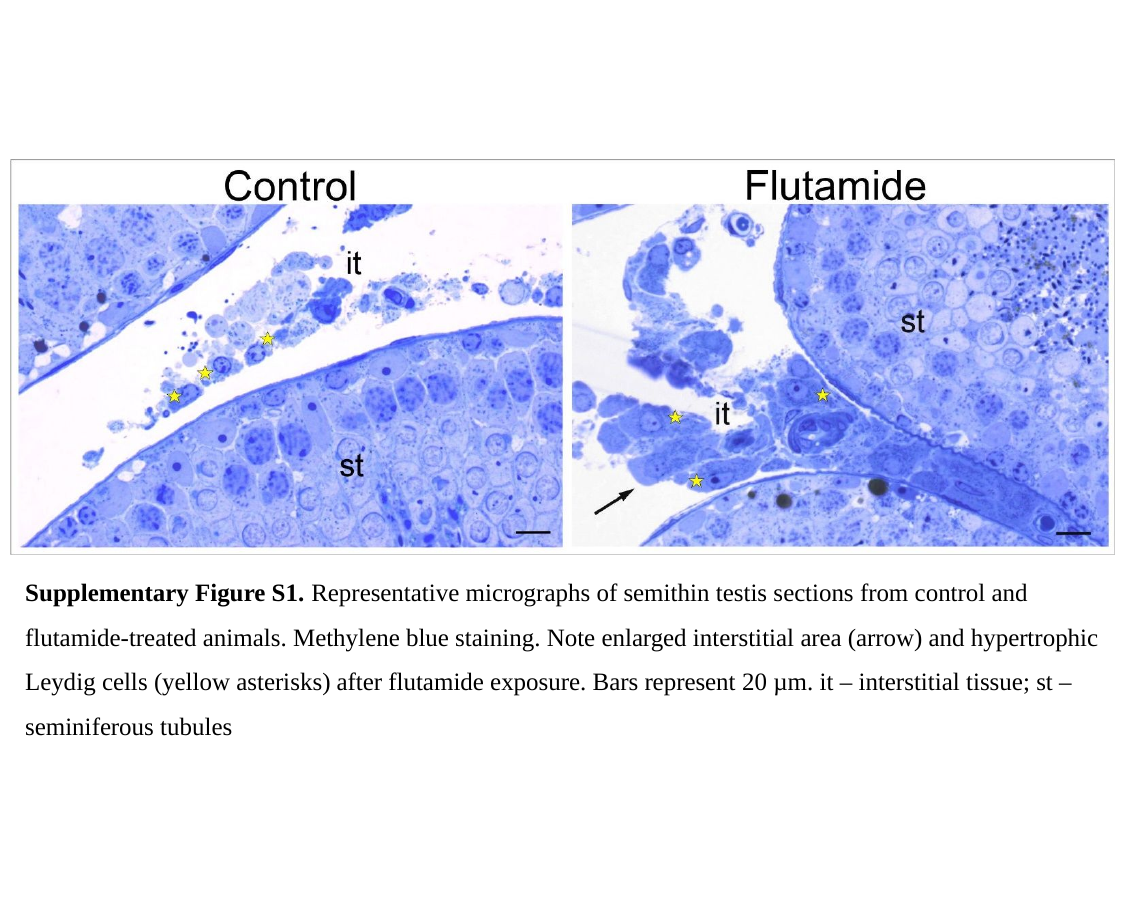

# Supplementary Figure S1. Representative micrographs of semithin testis sections from control and flutamide-treated animals. Methylene blue staining. Note enlarged interstitial area (arrow) and hypertrophic Leydig cells (yellow asterisks) after flutamide exposure. Bars represent 20 µm. it – interstitial tissue; st – seminiferous tubules

## Slide 3
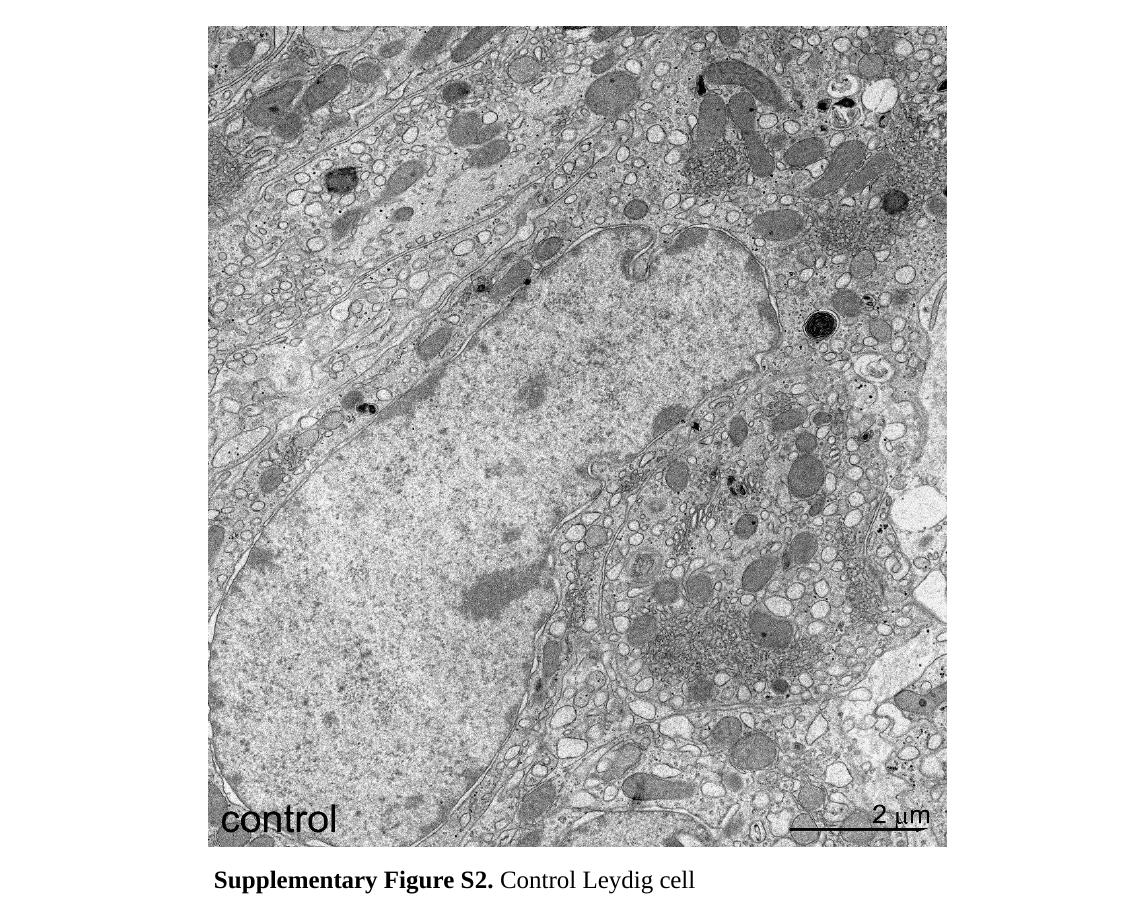

# Supplementary Figure S2. Control Leydig cell

## Slide 4
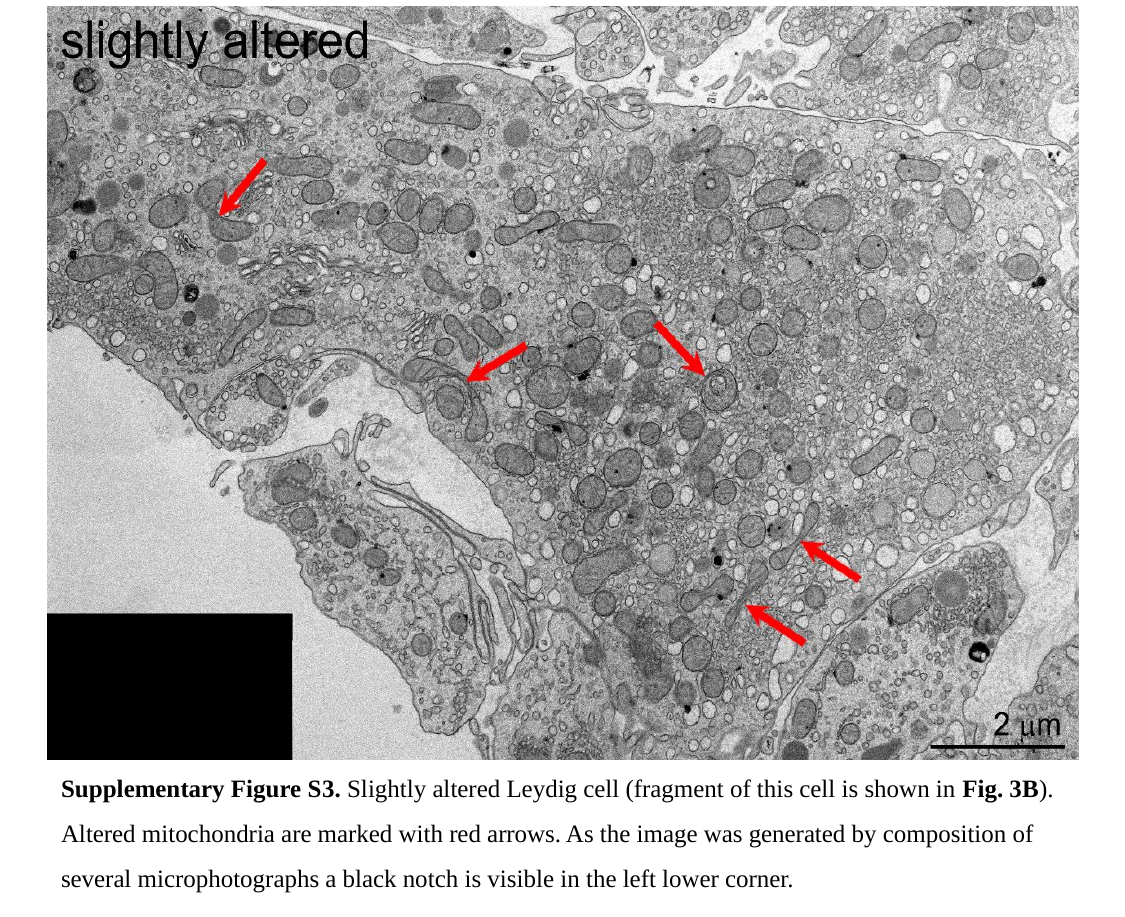

# Supplementary Figure S3. Slightly altered Leydig cell (fragment of this cell is shown in Fig. 3B). Altered mitochondria are marked with red arrows. As the image was generated by composition of several microphotographs a black notch is visible in the left lower corner.

## Slide 5
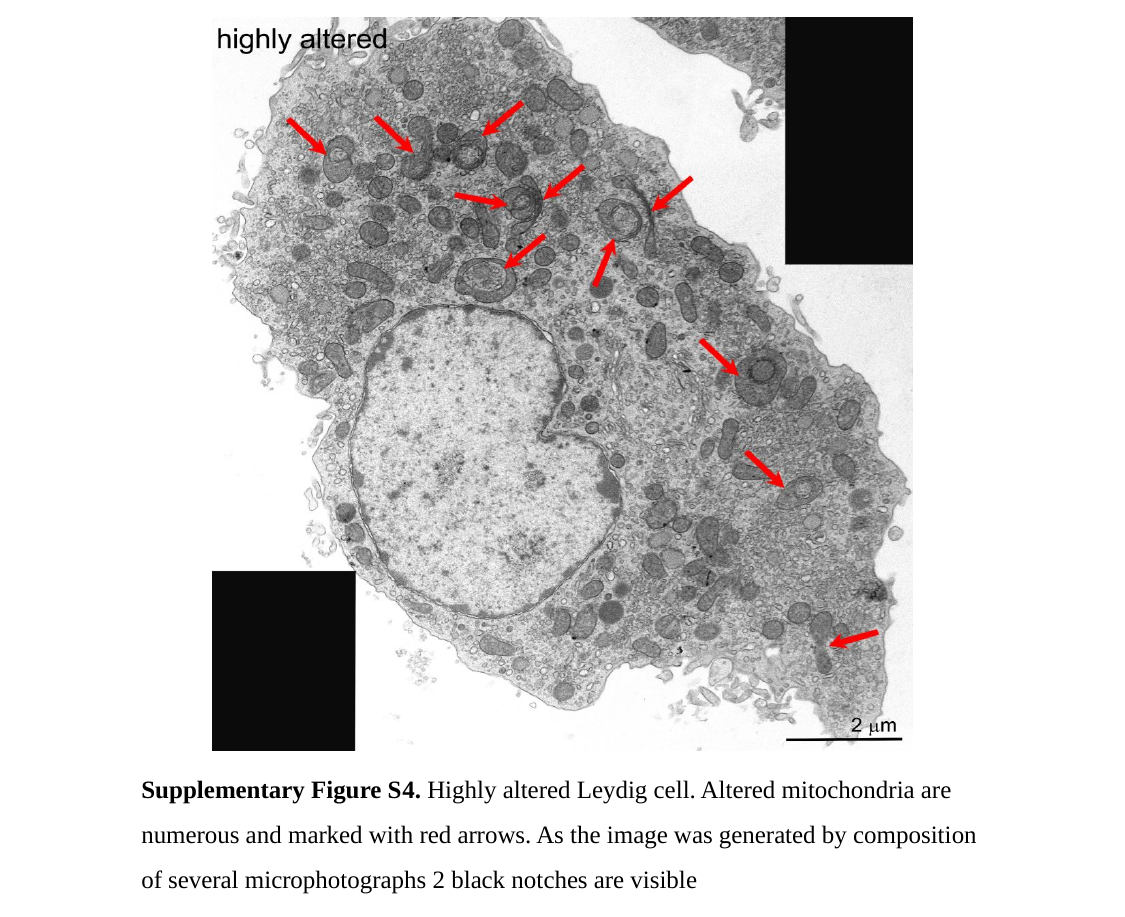

# Supplementary Figure S4. Highly altered Leydig cell. Altered mitochondria are numerous and marked with red arrows. As the image was generated by composition of several microphotographs 2 black notches are visible

## Slide 6
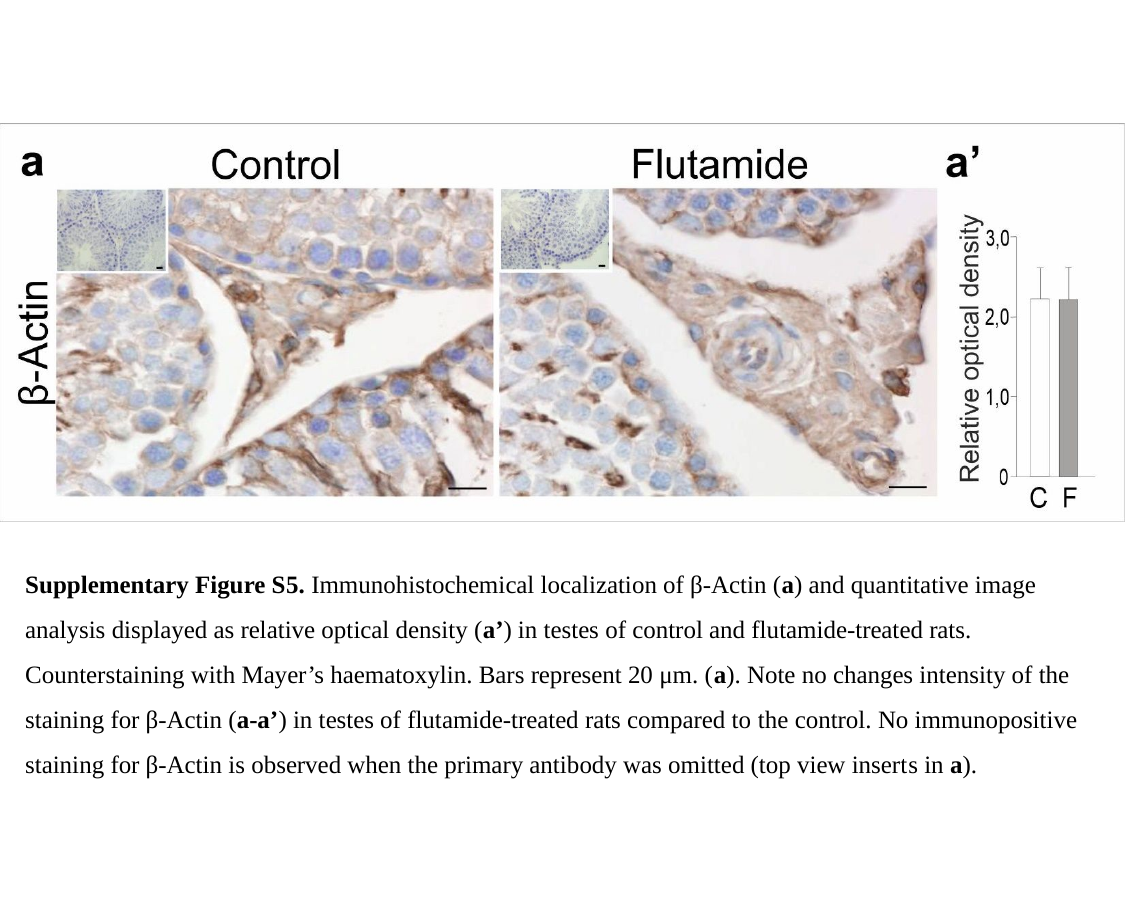

# Supplementary Figure S5. Immunohistochemical localization of β-Actin (a) and quantitative image analysis displayed as relative optical density (a’) in testes of control and flutamide-treated rats. Counterstaining with Mayer’s haematoxylin. Bars represent 20 μm. (a). Note no changes intensity of the staining for β-Actin (a-a’) in testes of flutamide-treated rats compared to the control. No immunopositive staining for β-Actin is observed when the primary antibody was omitted (top view inserts in a).

## Slide 7
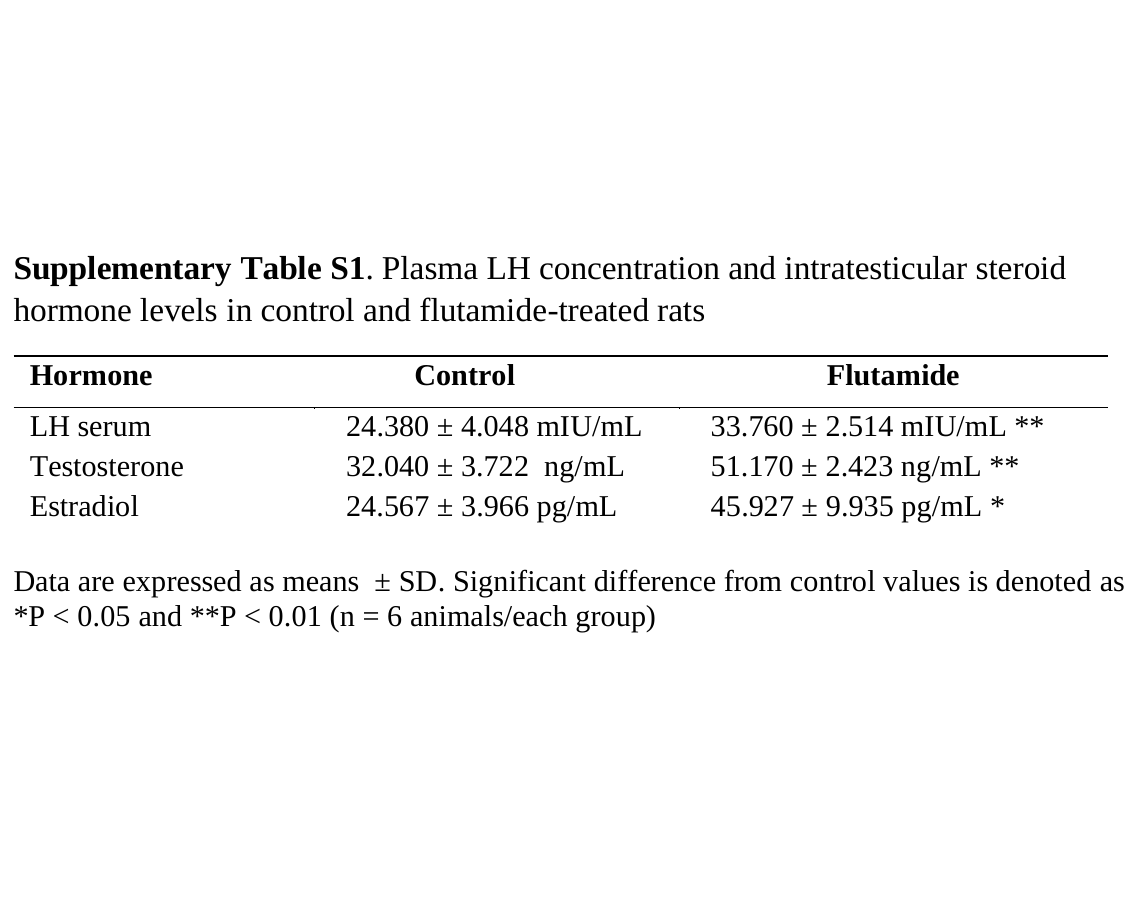

## Slide 8
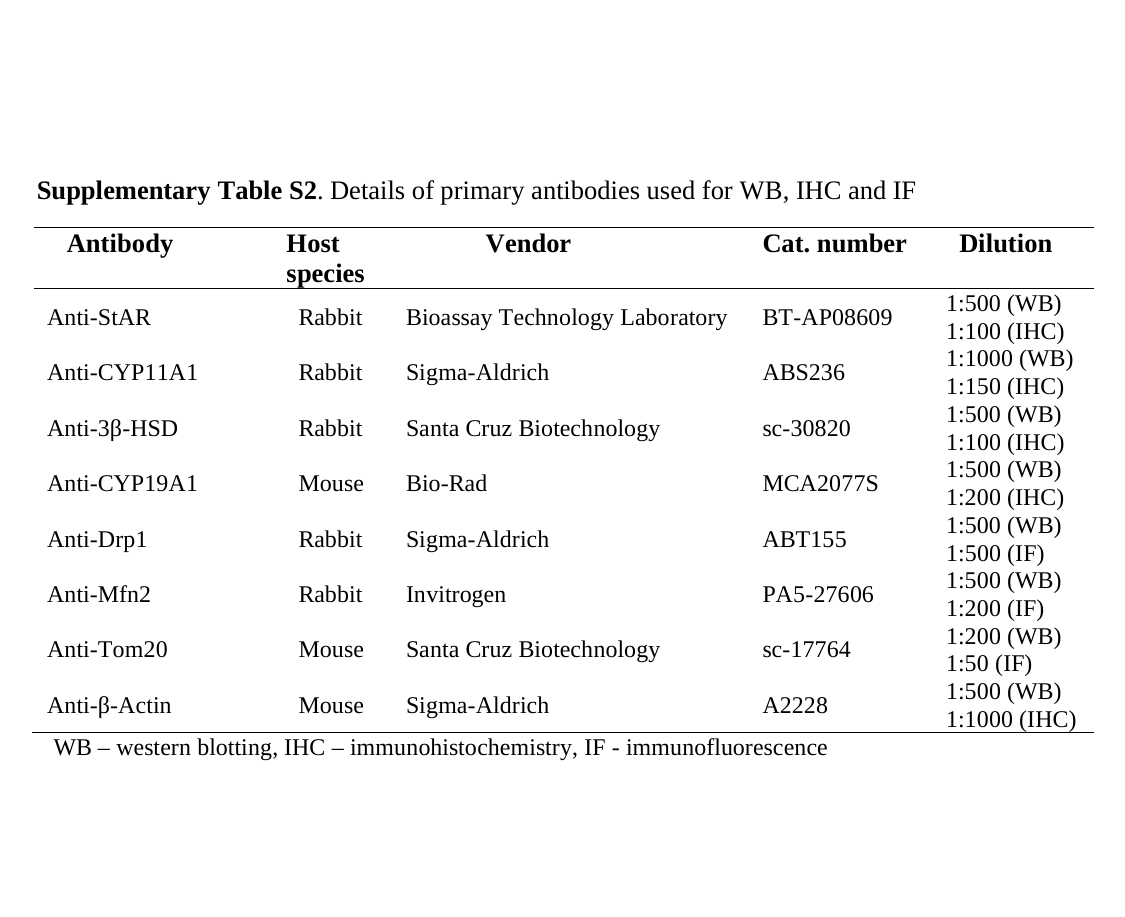

## Slide 9
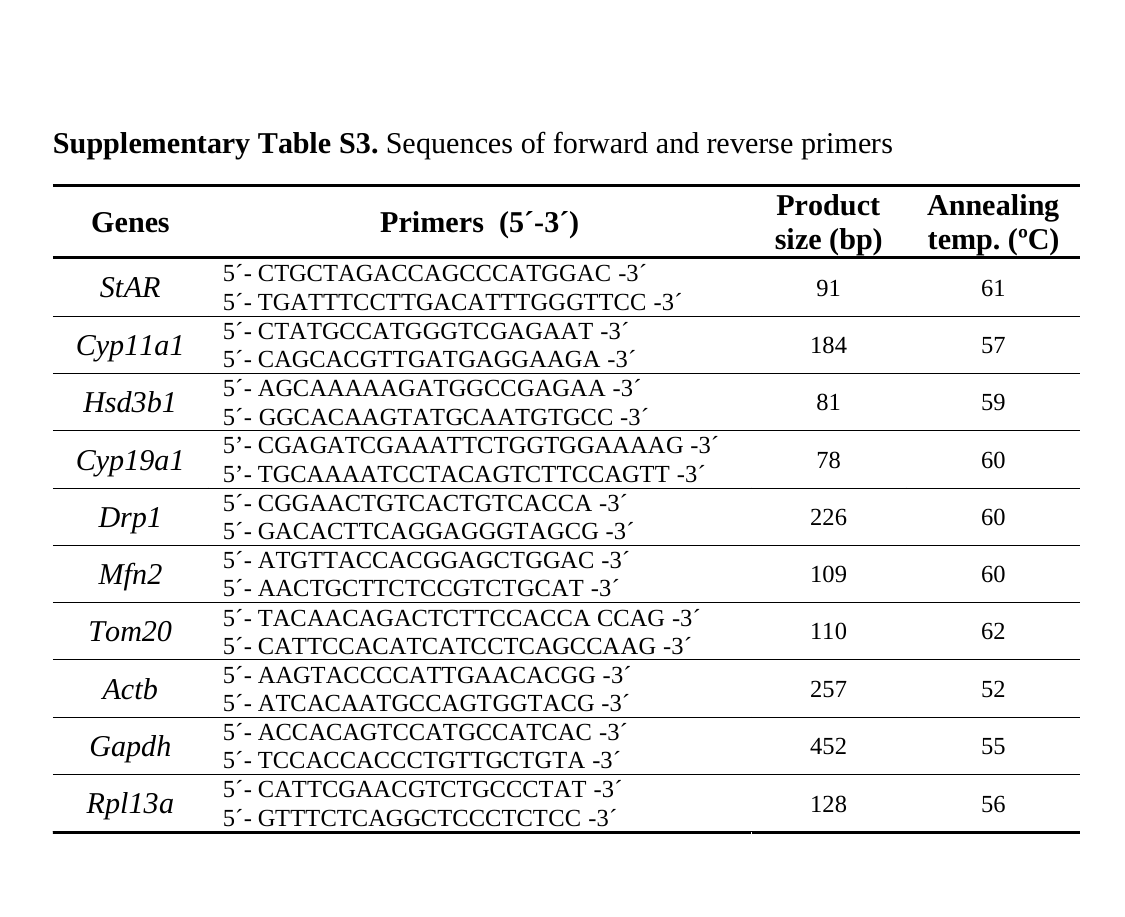

## Slide 10
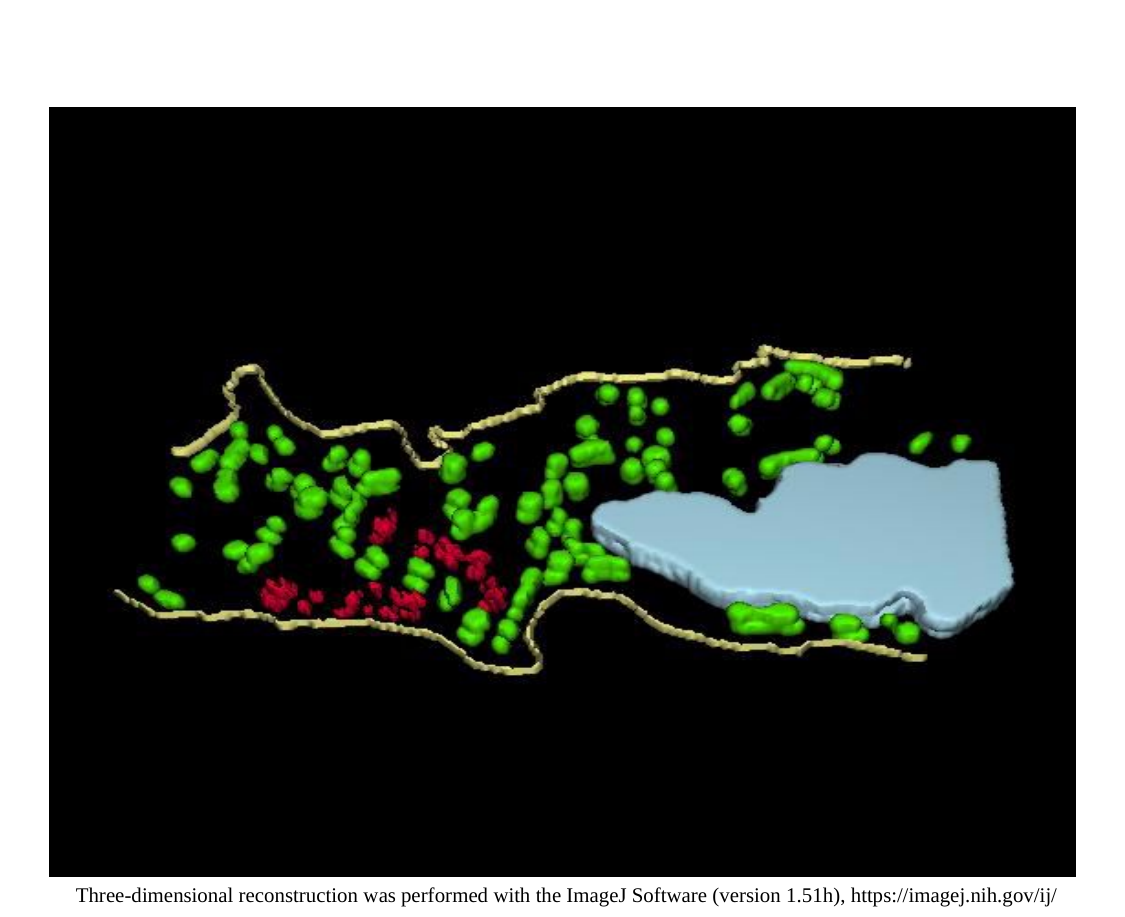

# Supplementary Video S1. Animated 3D reconstruction of control Leydig cell
Three-dimensional reconstruction was performed with the ImageJ Software (version 1.51h), https://imagej.nih.gov/ij/

## Slide 11
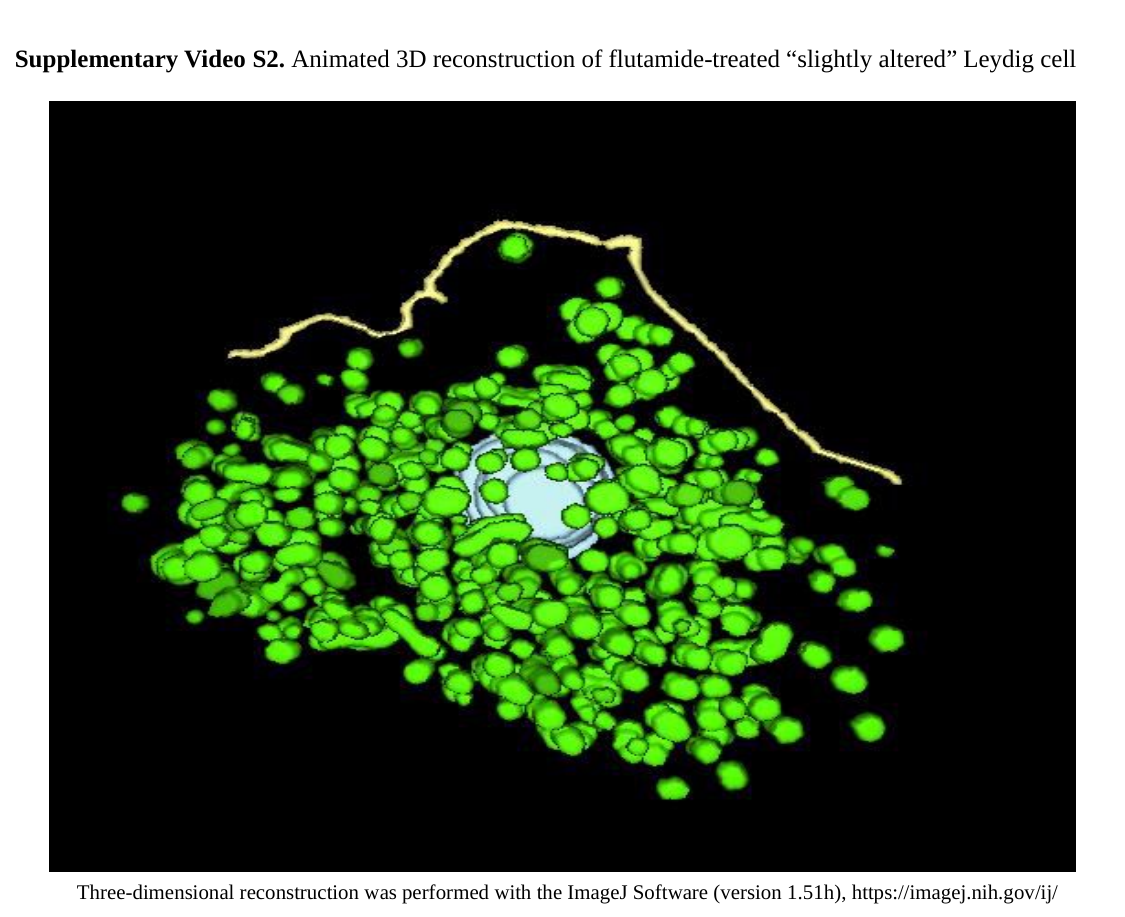

# Supplementary Video S2. Animated 3D reconstruction of flutamide-treated “slightly altered” Leydig cell
Three-dimensional reconstruction was performed with the ImageJ Software (version 1.51h), https://imagej.nih.gov/ij/

## Slide 12
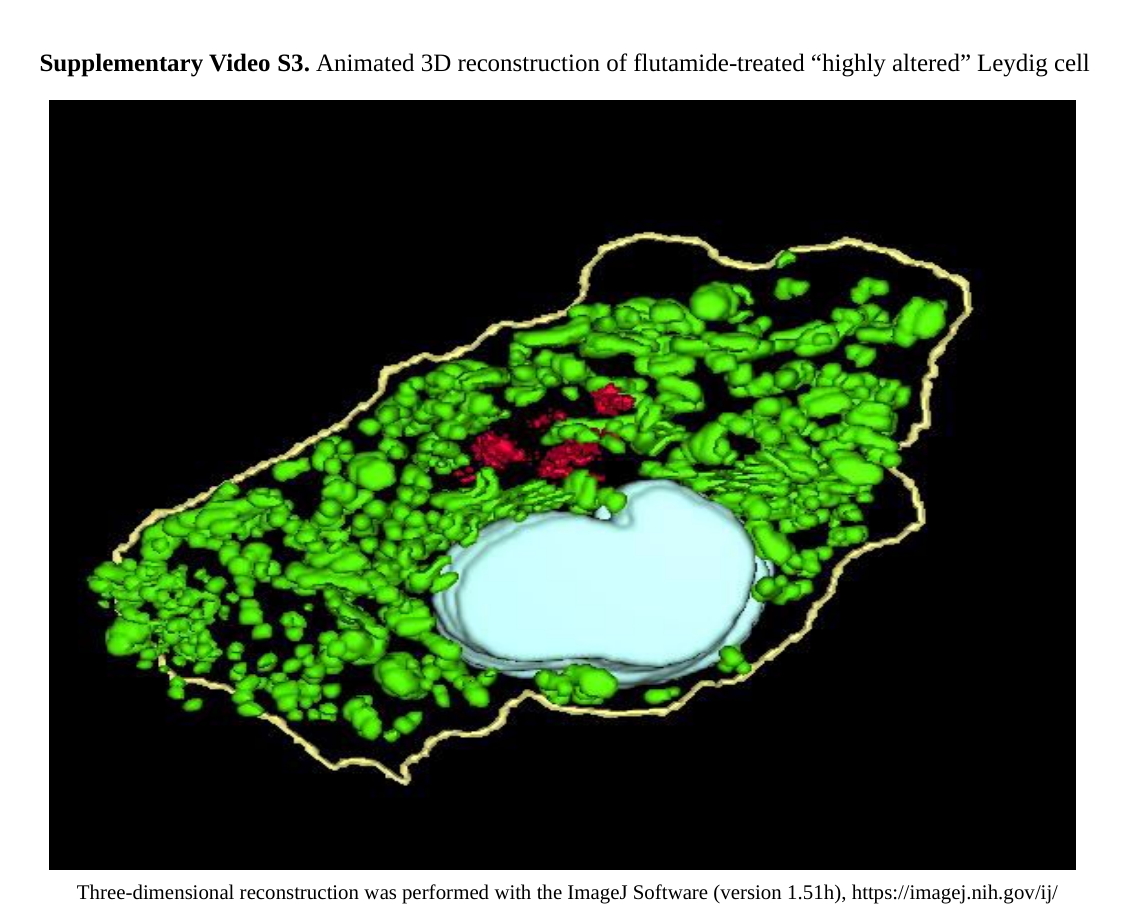

# Supplementary Video S3. Animated 3D reconstruction of flutamide-treated “highly altered” Leydig cell
Three-dimensional reconstruction was performed with the ImageJ Software (version 1.51h), https://imagej.nih.gov/ij/

## Slide 13
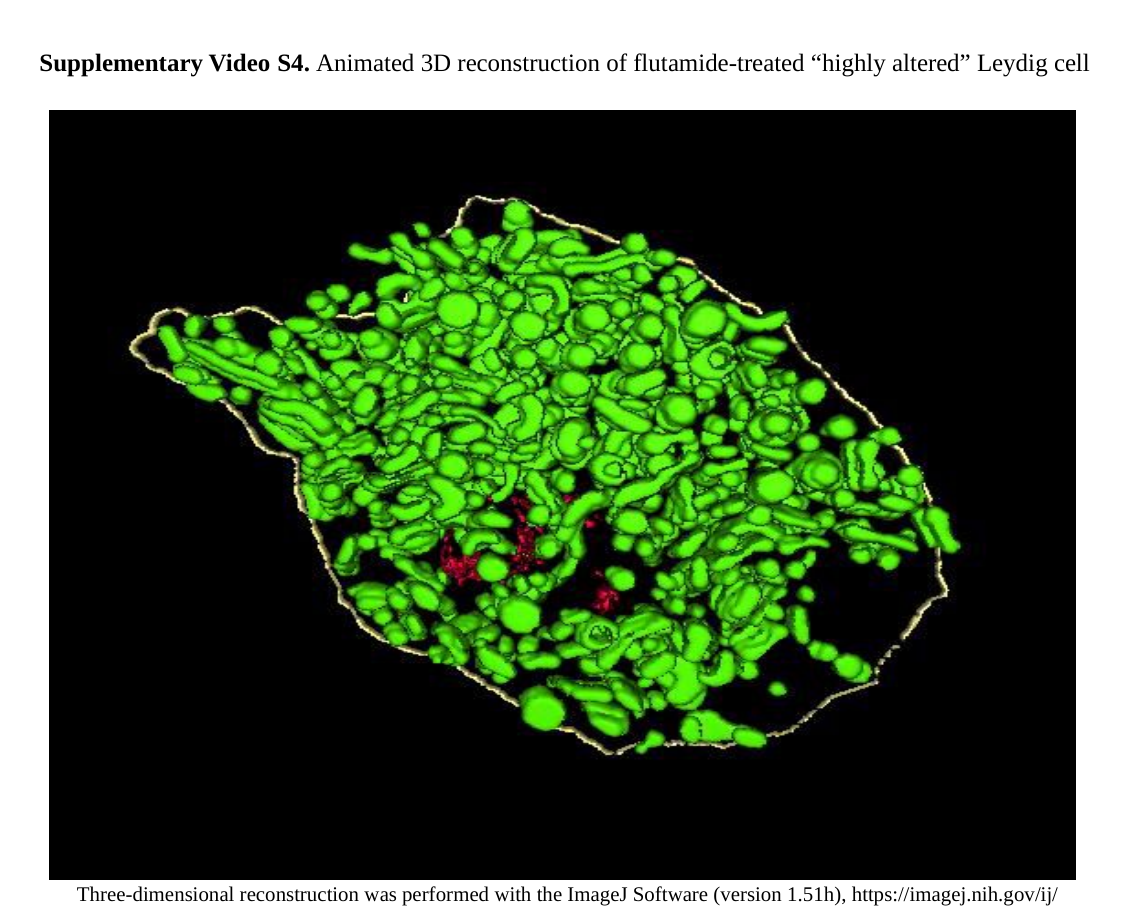

# Supplementary Video S4. Animated 3D reconstruction of flutamide-treated “highly altered” Leydig cell
Three-dimensional reconstruction was performed with the ImageJ Software (version 1.51h), https://imagej.nih.gov/ij/

## Slide 14
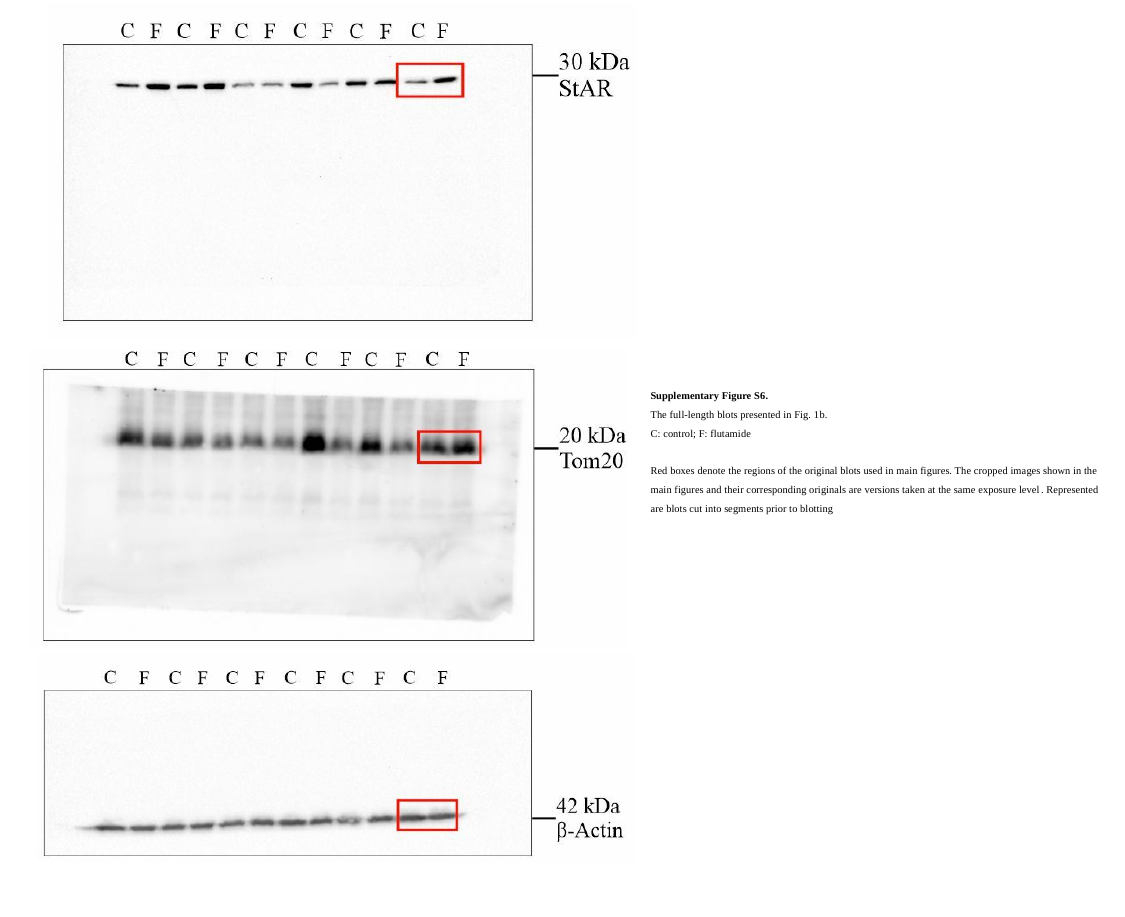

Supplementary Figure S6.
The full-length blots presented in Fig. 1b.
C: control; F: flutamide
Red boxes denote the regions of the original blots used in main figures. The cropped images shown in the main figures and their corresponding originals are versions taken at the same exposure level. Represented are blots cut into segments prior to blotting

## Slide 15
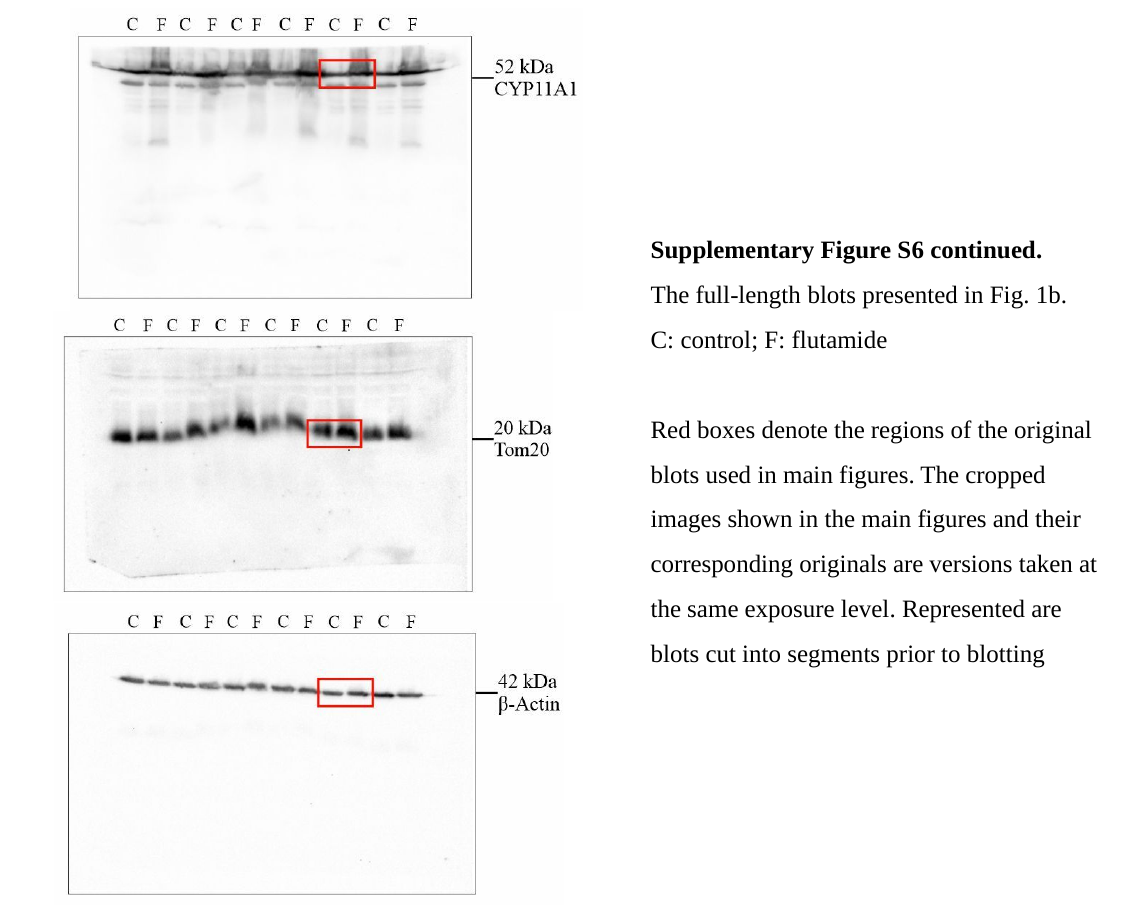

# Supplementary Figure S6 continued. The full-length blots presented in Fig. 1b. C: control; F: flutamide Red boxes denote the regions of the original blots used in main figures. The cropped images shown in the main figures and their corresponding originals are versions taken at the same exposure level. Represented are blots cut into segments prior to blotting

## Slide 16
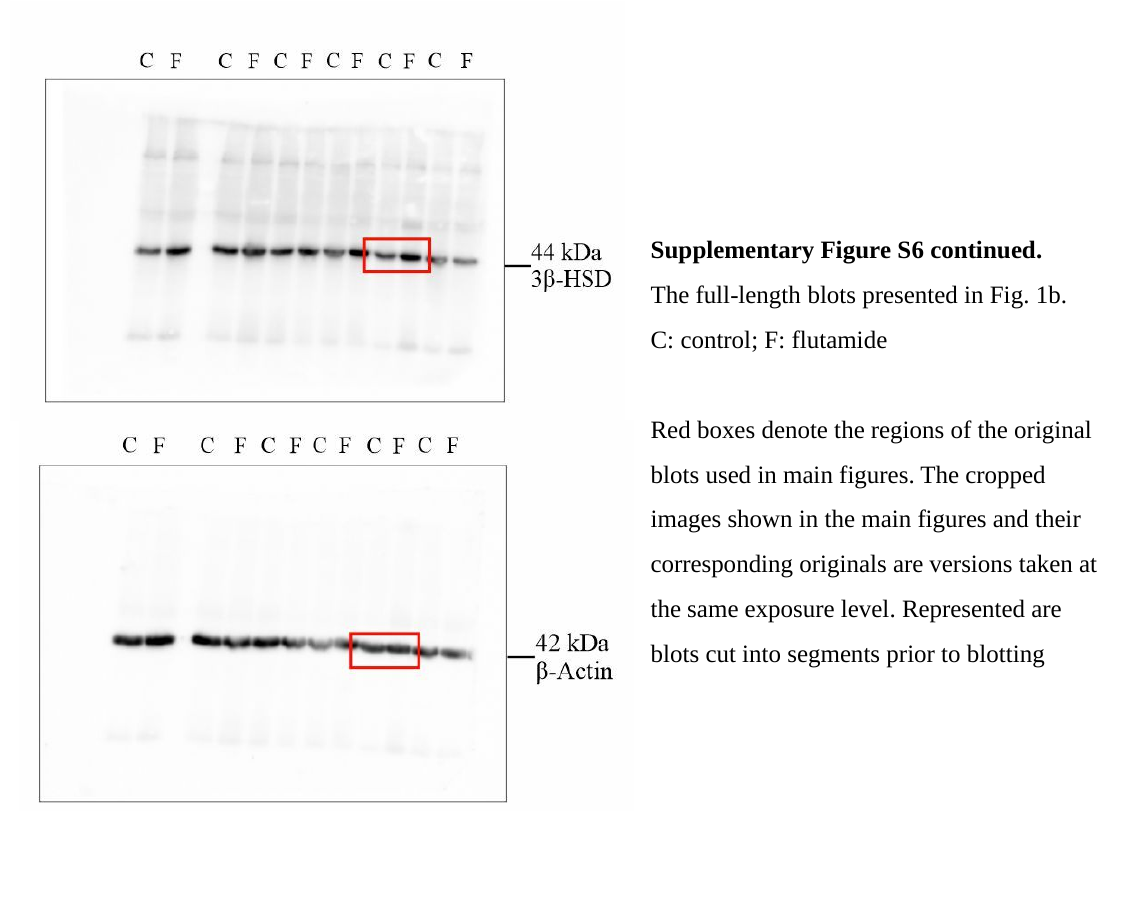

# Supplementary Figure S6 continued. The full-length blots presented in Fig. 1b. C: control; F: flutamide Red boxes denote the regions of the original blots used in main figures. The cropped images shown in the main figures and their corresponding originals are versions taken at the same exposure level. Represented are blots cut into segments prior to blotting

## Slide 17
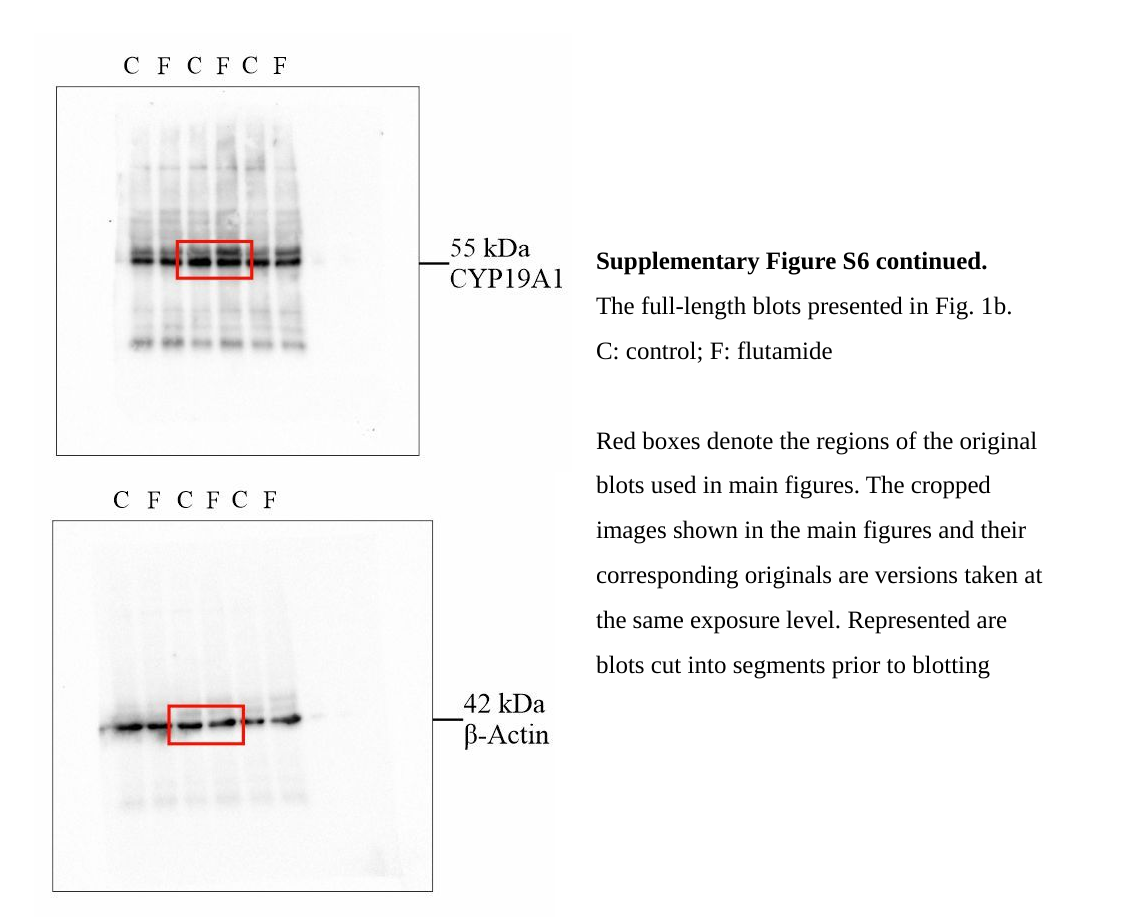

# Supplementary Figure S6 continued. The full-length blots presented in Fig. 1b. C: control; F: flutamide Red boxes denote the regions of the original blots used in main figures. The cropped images shown in the main figures and their corresponding originals are versions taken at the same exposure level. Represented are blots cut into segments prior to blotting

## Slide 18
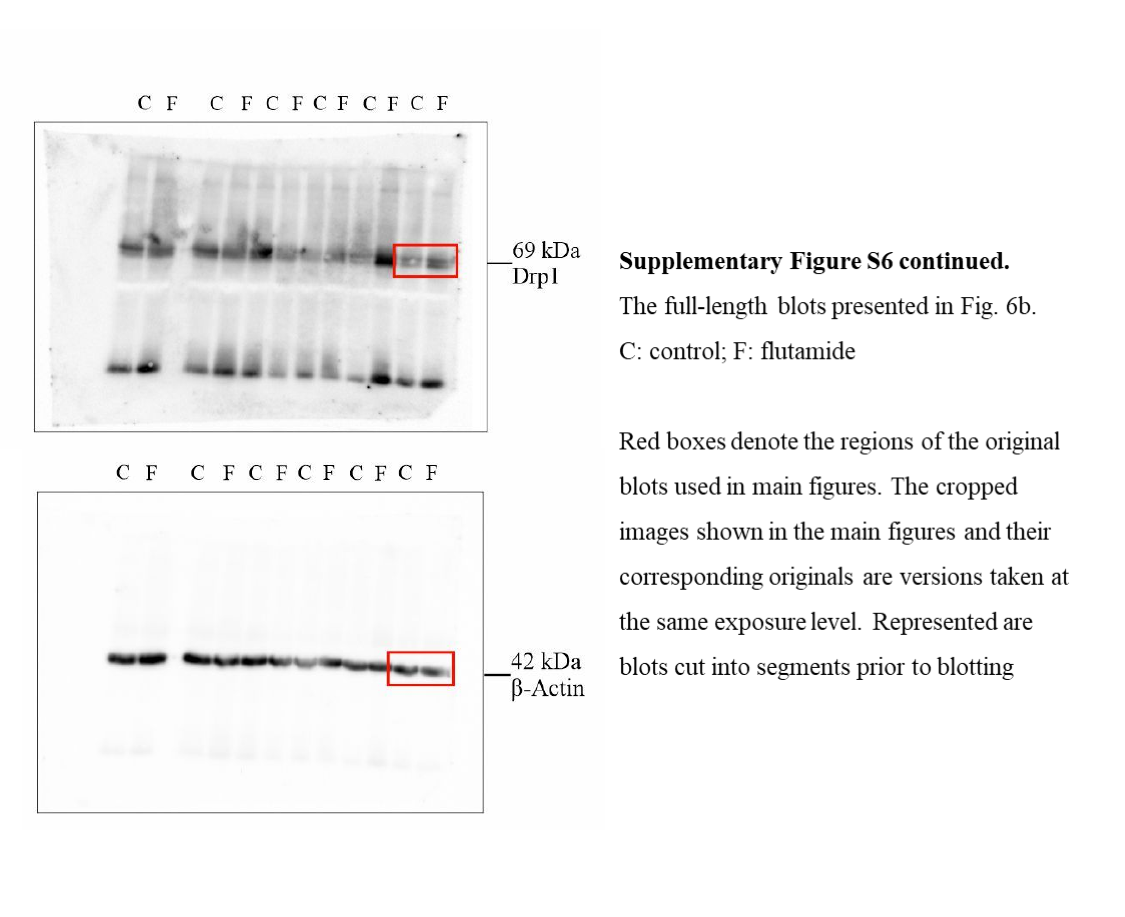

## Slide 19
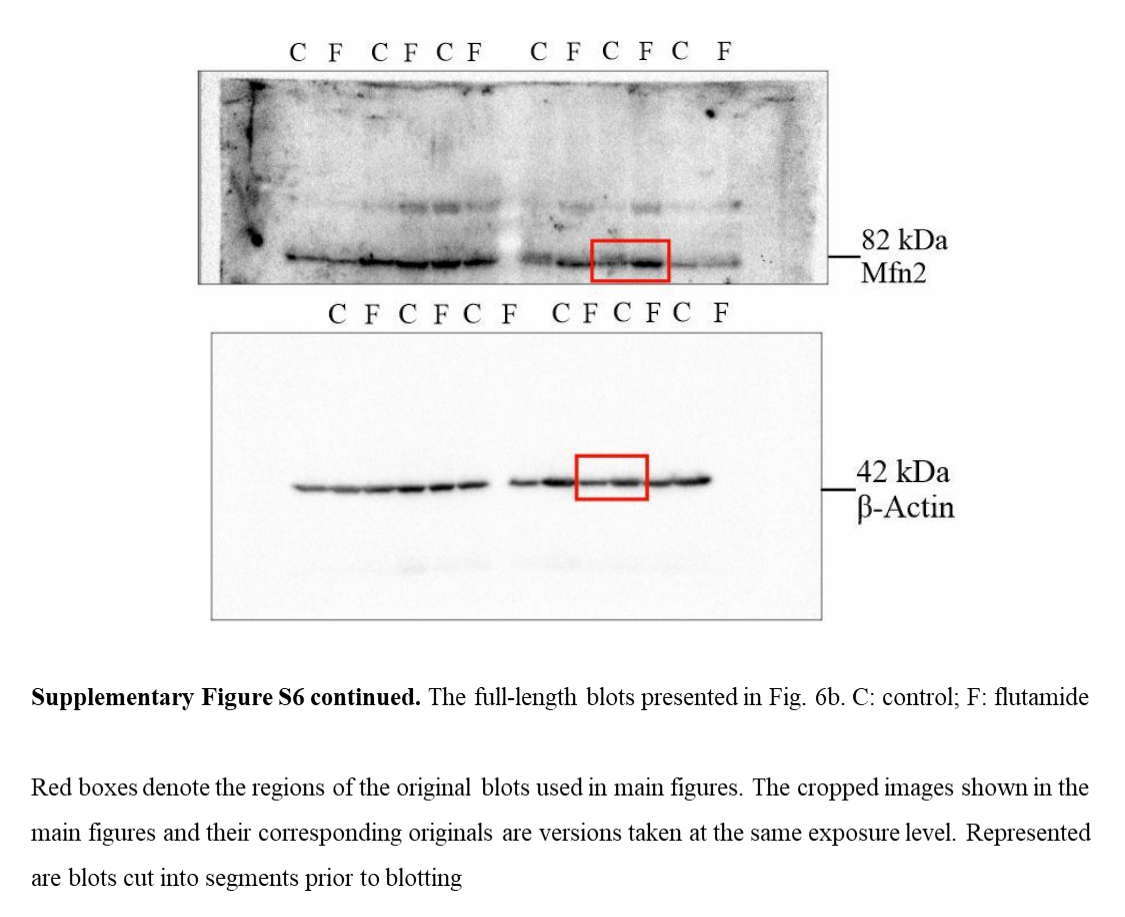

## Slide 20
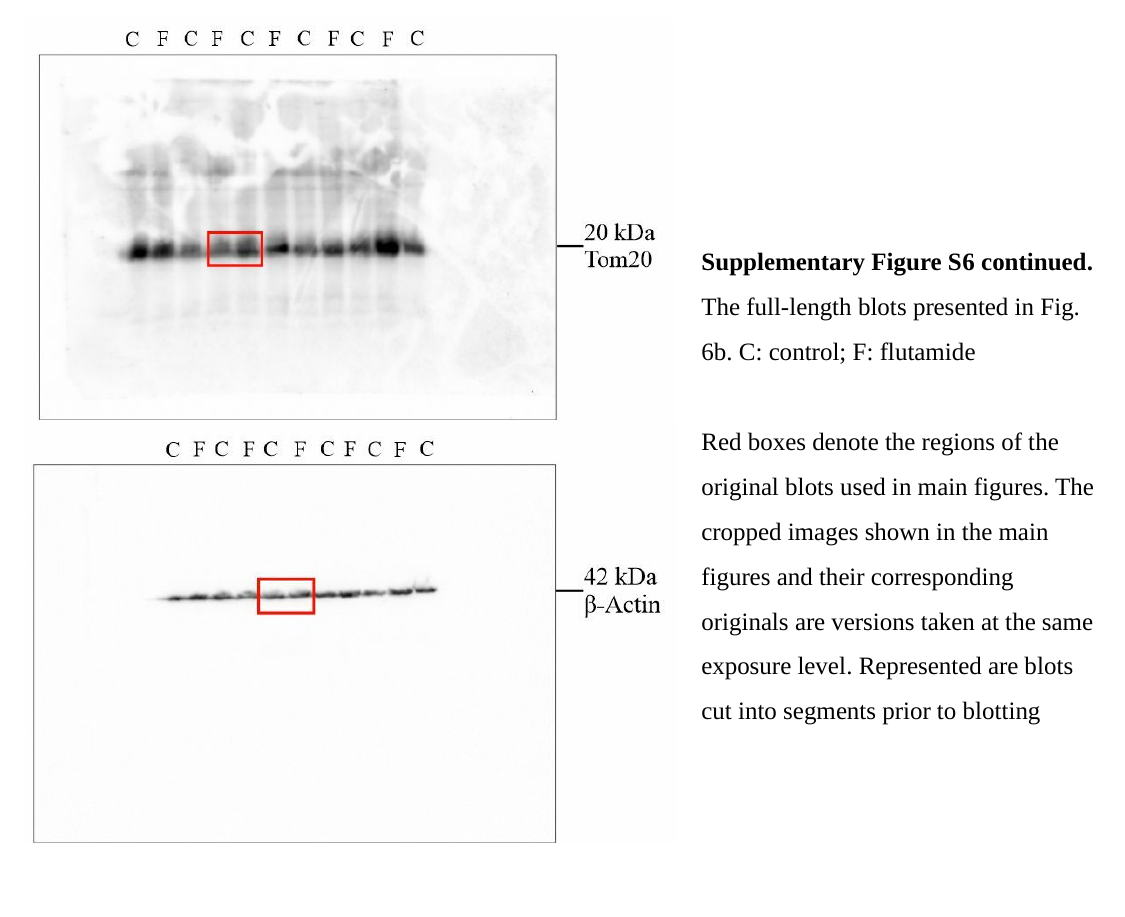

# Supplementary Figure S6 continued. The full-length blots presented in Fig. 6b. C: control; F: flutamide Red boxes denote the regions of the original blots used in main figures. The cropped images shown in the main figures and their corresponding originals are versions taken at the same exposure level. Represented are blots cut into segments prior to blotting

## Slide 21
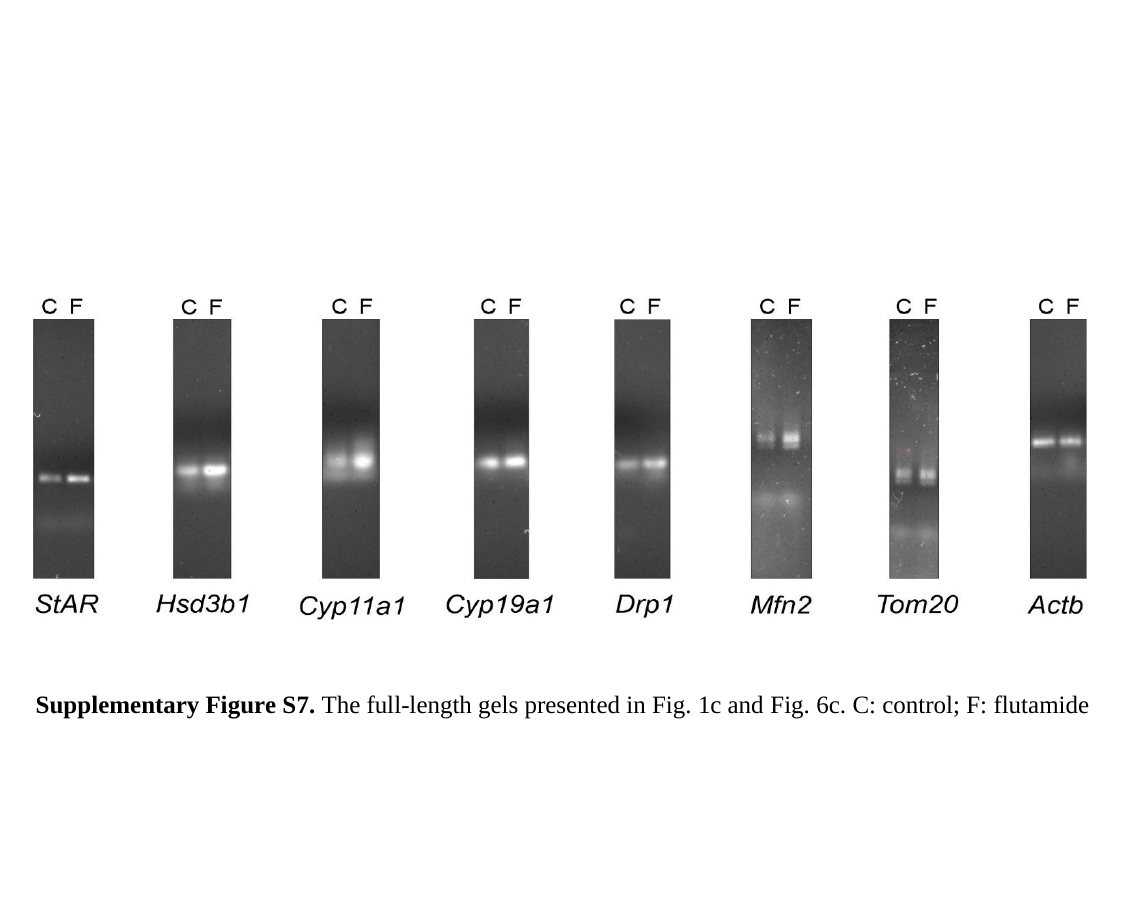

# Supplementary Figure S7. The full-length gels presented in Fig. 1c and Fig. 6c. C: control; F: flutamide
